# Fulvestrant 500 mg Versus Anastrozole 1 mg for the First-Line Treatment of Advanced Breast Cancer: Overall Survival Analysis From the Phase II FIRST Study

**DOI:** 10.1200/JCO.2015.61.5831

**Published:** 2015-09-14

**Authors:** Matthew J. Ellis, Antonio Llombart-Cussac, David Feltl, John A. Dewar, Marek Jasiówka, Nicola Hewson, Yuri Rukazenkov, John F.R. Robertson

**Affiliations:** Matthew J. Ellis, Baylor College of Medicine, Houston, TX; Antonio Llombart-Cussac, Hospital Arnau de Vilanova, Lérida, Spain; David Feltl, FNsP Ostrava, Ostrava-Poruba, Czech Republic; John A. Dewar, Ninewells Hospital and Medical School, Dundee; Nicola Hewson and Yuri Rukazenkov, AstraZeneca Pharmaceuticals, Macclesfield; John F.R. Robertson, University of Nottingham, Derby, United Kingdom; and Marek Jasiówka, Instytut im Marii Skłodowskiej-Curie, Kraków, Poland.

## Abstract

**Purpose:**

To compare overall survival (OS) for fulvestrant 500 mg versus anastrozole as first-line endocrine therapy for advanced breast cancer.

**Patients and Methods:**

The Fulvestrant First-Line Study Comparing Endocrine Treatments (FIRST) was a phase II, randomized, open-label, multicenter trial. Postmenopausal women with estrogen receptor–positive, locally advanced/metastatic breast cancer who had no previous therapy for advanced disease received either fulvestrant 500 mg (days 0, 14, 28, and every 28 days thereafter) or anastrozole 1 mg (daily). The primary end point (clinical benefit rate [72.5% and 67.0%]) and a follow-up analysis (median time to progression [23.4 months and 13.1 months]) have been reported previously for fulvestrant 500 mg and anastrozole, respectively. Subsequently, the protocol was amended to assess OS by unadjusted log-rank test after approximately 65% of patients had died. Treatment effect on OS across several subgroups was examined. Tolerability was evaluated by adverse event monitoring.

**Results:**

In total, 205 patients were randomly assigned (fulvestrant 500 mg, n = 102; anastrozole, n = 103). At data cutoff, 61.8% (fulvestrant 500 mg, n = 63) and 71.8% (anastrozole, n = 74) had died. The hazard ratio (95% CI) for OS with fulvestrant 500 mg versus anastrozole was 0.70 (0.50 to 0.98; *P* = .04; median OS, 54.1 months *v* 48.4 months). Treatment effects seemed generally consistent across the subgroups analyzed. No new safety issues were observed.

**Conclusion:**

There are several limitations of this OS analysis, including that it was not planned in the original protocol but instead was added after time-to-progression results were analyzed, and that not all patients participated in additional OS follow-up. However, the present results suggest fulvestrant 500 mg extends OS versus anastrozole. This finding now awaits prospective confirmation in the larger phase III FALCON (Fulvestrant and Anastrozole Compared in Hormonal Therapy Naïve Advanced Breast Cancer) trial (ClinicalTrials.gov identifier: NCT01602380).

## INTRODUCTION

Tamoxifen and third-generation aromatase inhibitors (AIs), such as anastrozole, exemestane, and letrozole are established first-line endocrine therapies for the treatment of postmenopausal women with estrogen receptor (ER) –positive, advanced breast cancer.^1–3^ Given the high prevalence of resistance to AI therapy, multiple treatment options with distinct mechanisms of action are desirable.^4^

Fulvestrant, a 17β-estradiol analog, is a selective ER antagonist that suppresses estrogen signaling by binding to ER and inducing a conformational change.^5,6^ Dimerization is subsequently blocked, triggering accelerated degradation and downregulation of the ER protein.^5^ Fulvestrant exhibits lack of cross-reactivity with tamoxifen. Consequently, patients whose disease progresses on fulvestrant may retain sensitivity to treatment with further endocrine therapies.^7,8^ The clinical efficacy of fulvestrant was initially demonstrated in two phase III trials that compared fulvestrant 250 mg per month with anastrozole 1 mg daily as a second-line therapy for advanced breast cancer.^9,10^ A combined analysis of these trials demonstrated that time to progression (TTP) with fulvestrant 250 mg was noninferior to anastrozole.^11^

Fulvestrant 250 mg was not proven to be superior to tamoxifen in a double-blind, randomized trial.^12^ This finding was unexpected given the superiority of anastrozole over tamoxifen^13^ and the comparable efficacy of anastrozole and fulvestrant 250 mg as second-line therapy.^11^ Pharmacokinetic modeling, as well as observations made during early clinical studies,^11^ suggested the efficacy of fulvestrant could be improved with use of a higher dose, which led to the development of a dosage regimen of fulvestrant 500 mg, including a loading dose component to reduce the time to reach steady-state plasma levels. Subsequently, the phase III Comparison of Faslodex in Recurrent or Metastatic Breast Cancer (CONFIRM) trial found that fulvestrant 500 mg was associated with improved progression-free survival (PFS) and overall survival (OS) compared with the 250-mg dose in patients who experienced disease recurrence or progression after previous endocrine therapy.^14,15^

The Fulvestrant First-Line Study Comparing Endocrine Treatments (FIRST) was a phase II, randomized, open-label, multicenter trial that also used the fulvestrant 500-mg dose regimen, comparing efficacy and safety with anastrozole in the first-line setting. The primary end point of clinical benefit rate was noninferior for fulvestrant 500 mg compared with anastrozole,^16^ with both treatments demonstrating similar, well-tolerated safety profiles. A follow-up analysis, performed because only 35.6% of patients experienced disease progression at the time of the primary analysis, reported a hazard ratio (HR) of TTP for fulvestrant 500 mg versus anastrozole of 0.66 with a 95% CI of 0.47 to 0.92 (*P* = .01; median TTP, 23.4 months *v* 13.1 months). No additional safety issues were reported.^17^ Given the improvement in TTP observed during fulvestrant 500 mg treatment compared with anastrozole in this phase II trial, a subsequent protocol amendment was made to address whether this apparent extension in disease control would translate into an improvement in OS.

## PATIENTS AND METHODS

### Study Design and Participants

FIRST was a phase II, randomized, open-label, multicenter, parallel-group trial comparing fulvestrant 500 mg with anastrozole 1 mg. Postmenopausal women with ER-positive locally advanced or metastatic breast cancer who had not received any previous systemic therapy for locally advanced or metastatic disease were included. Patients were permitted to have received previous endocrine therapy for early disease, providing this had been completed more than 12 months before random assignment. This trial was conducted in accordance with the Declaration of Helsinki, was consistent with the International Conference on Harmonisation–Good Clinical Practice guidelines, and is registered with Clinicaltrials.gov. All patients provided written, informed consent. Full details of this trial have been reported previously.^16,17^

### Random Assignment and Procedures

Eligible patients were randomly assigned sequentially 1:1 to either fulvestrant 500 mg (administered intramuscularly on days 0, 14, 28, and every 28 days thereafter) or anastrozole 1 mg (administered orally once per day). The data cutoff for the primary analysis was 6 months after the last patient was randomly assigned. On disease progression or after data cutoff for the primary analysis, all patients entered a follow-up phase after a protocol amendment for an analysis of TTP. The TTP follow-up required a questionnaire to be completed for each patient 12 months after the patient entered the follow-up phase and every 12 months thereafter for patients continuing to receive randomized treatment. After the TTP analysis was performed, a further protocol amendment was developed to enter patients into an optional follow-up phase to establish OS. To ensure sufficient maturity, the OS analysis was planned for when approximately 65% of patients had died. Patients who did not contribute additional data to the follow-up extension were right-censored at the last known date they were alive, and their data until this point were included in the analysis. Sites were invited to request written consent from patients for the collection of additional data. Patients were contacted every 3 months until the first of the following events: death, patient withdrawal, data cutoff was reached, or the patient was lost to follow-up. Patients with a last known survival status of alive were contacted within 2 weeks of data cutoff to ensure they were still alive.

### Outcomes

The primary study end point was clinical benefit rate; secondary end points included objective response rate, TTP, duration of clinical benefit, and duration of response. These primary and secondary end points have been reported previously.^16,17^

The follow-up analysis assessed OS, defined as the time from being randomly assigned to death from any cause. A log-rank test (unadjusted model with treatment factor only) was performed for the primary analysis of OS. HRs with 95% CIs were used to compare fulvestrant 500 mg with anastrozole; no adjustments were made for multiplicity. A statistical significance level of .05 was used to indicate a difference in OS between the treatment groups. For patients for whom follow-up responses could not be obtained, data were censored at the date the patient was last known to be alive.

Exploratory subgroup analyses were conducted using the log-rank test to compare OS for the following prespecified patient subgroups: less than 65 years of age versus 65 years of age or greater; not positive for both ER and progesterone receptor versus positive for both ER and progesterone receptor; no visceral involvement versus visceral involvement; no previous chemotherapy versus previous adjuvant chemotherapy; no measurable disease versus measurable disease; and no previous endocrine therapy versus previous endocrine therapy.

Two sensitivity analyses were performed to examine any potential impact of nonparticipation on OS results: a Kaplan-Meier OS analysis was performed in which the censoring indicator was reversed; and baseline covariates were assessed for patients censored greater than 3 months before data cutoff and for those censored 3 months or less before data cutoff, which corresponds to patients who did not participate in the OS follow-up and to those who did, respectively.

Tolerability was assessed by serious adverse event (SAE) monitoring. All SAEs were coded in compliance with the Medical Dictionary for Regulatory Activities and recorded in an internal AstraZeneca database for evaluation. SAEs were monitored for up to 8 weeks after the last dose of fulvestrant 500 mg or for 30 days after the last dose of anastrozole.

## RESULTS

In total, 205 patients were randomly assigned to receive fulvestrant 500 mg (n = 102) or anastrozole 1 mg (n = 103) at 62 centers in nine countries (Brazil, Bulgaria, the Czech Republic, France, Italy, Poland, Spain, the United Kingdom, and the United States).

Baseline characteristics and patient demographics were similar between the treatment groups as reported previously.^16^ The proportion of patients who had not received previous endocrine treatment for early disease was similar for the fulvestrant 500 mg and anastrozole treatment groups (71.6% and 77.7% of patients at baseline, respectively). Of those that did, almost all had received tamoxifen exclusively. Of the 205 randomly assigned patients, 35 (16 in the fulvestrant 500 mg group and 19 in the anastrozole group) did not participate in the OS follow-up phase and were censored at the date they were last known to be alive; for these patients, data until this time are included in the OS analysis, and thus all patients contributed data to the analysis. The majority of the nonparticipating patients (n = 20) did not contribute additional data because they attended centers that declined to contribute to the OS follow-up phase. An additional 15 individual patients from nine participating centers did not consent to follow-up. No patients participating in the OS phase were lost to follow-up, and the survival status at data cutoff was known for all patients consenting to the OS follow-up.

### Efficacy

**Fig 1.**
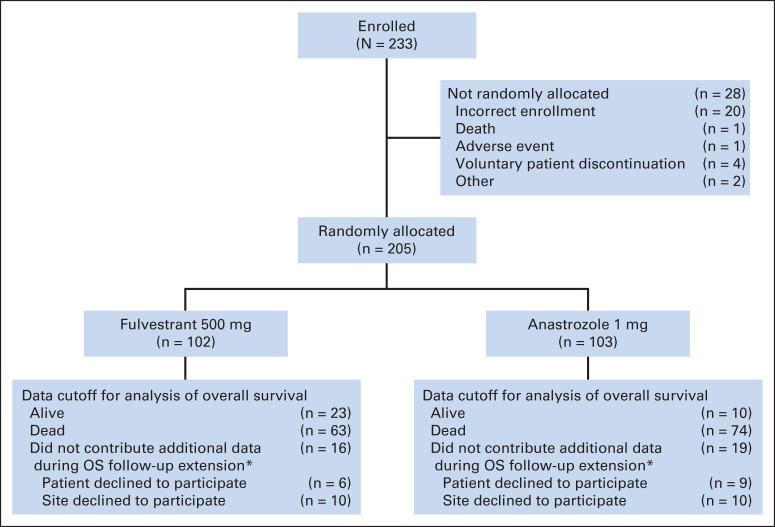
Study overview. (*) These patients were right censored at the time of their last known date alive, and data until this point were used in the overall survival (OS) analysis.

**Fig 2.**
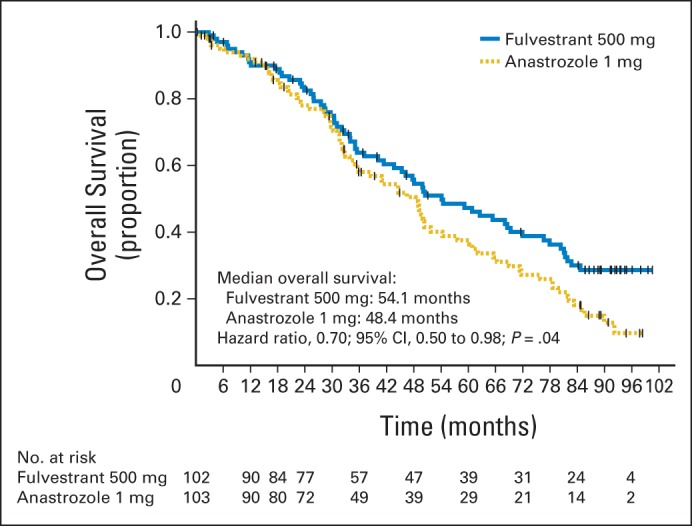
Kaplan-Meier plot of overall survival.

**Fig 3.**
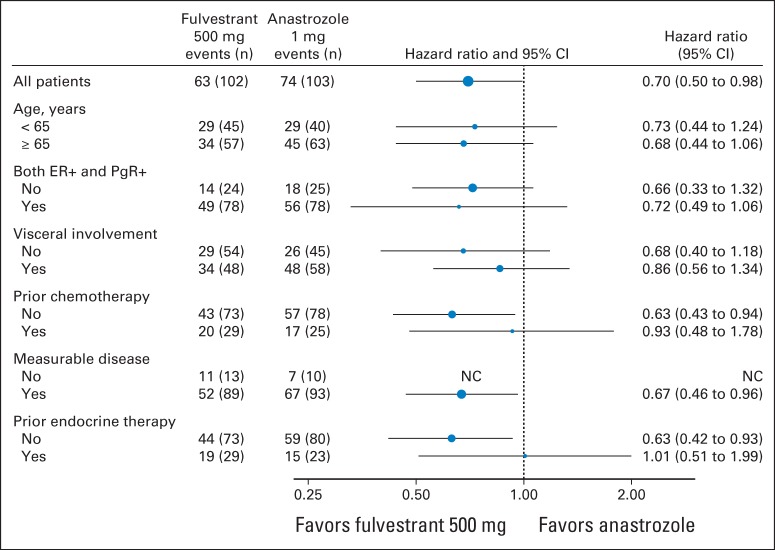
Overall survival subgroup analysis. ER+, estrogen receptor positive; NC, not calculable; PgR+, progesterone receptor positive.

At the time of the follow-up analysis for OS, 63 of 102 patients in the fulvestrant 500 mg group (61.8%) and 74 of 103 patients in the anastrozole group (71.8%) were known to have died (Fig 1). The primary analysis of OS was improved in the fulvestrant 500 mg group compared with anastrozole 1 mg; the HR was 0.70 (95% CI, 0.50 to 0.98; log-rank test *P* = .04; median OS, 54.1 months *v* 48.4 months; Fig 2). The HR for fulvestrant 500 mg versus anastrozole was found to be generally consistent across all subgroup analyses (Fig 3). At 3 years, 64% (fulvestrant 500 mg) and 58% (anastrozole) of patients were event free; at 5 years, the equivalent values were 47% and 38%.

### Sensitivity Analyses

There were no important differences between the treatment groups in time to censoring (data not shown). Furthermore, when key baseline covariates for patients censored within the last 3 months before data cutoff and for those censored more than 3 months before data cutoff were summarized, there were no important differences between treatment groups, indicating that the results were not caused by differences between patients who did and did not consent to OS follow-up (Table 1).

**Table 1.** Baseline Covariates and Subgroups by Patients Censored ≥ 3 Months and ≤ 3 Months Before DCO

Subgroup	No. of Patients (%)			
	Censored > 3 Months Before DCO		Censored ≤ 3 Months Before DCO	
	Fulvestrant 500 mg (n = 16)	Anastrozole 1 mg (n = 19)	Fulvestrant 500 mg (n = 23)	Anastrozole 1 mg (n = 10)
Age, years				
< 65	5 (31.3)	7 (36.8)	11 (47.8)	4 (40.0)
≥ 65	11 (68.8)	12 (63.2)	12 (52.2)	6 (60.0)
Receptor status at diagnosis				
Not both ER+ and PgR+	6 (37.5)	5 (26.3)	4 (17.4)	2 (20.0)
Both ER+ and PgR+	10 (62.5)	14 (73.7)	19 (82.6)	8 (80.0)
Visceral involvement				
No	9 (56.3)	11 (57.9)	16 (69.6)	8 (80.0)
Yes	7 (43.8)	8 (42.1)	7 (30.4)	2 (20.0)
Previous chemotherapy				
No	11 (68.8)	13 (68.4)	19 (82.6)	8 (80.0)
Yes	5 (31.3)	6 (31.6)	4 (17.4)	2 (20.0)
Measurable disease at diagnosis				
No	1 (6.3)	3 (15.8)	1 (4.3)	0
Yes	15 (93.8)	16 (84.2)	22 (95.7)	10 (100.0)
Previous endocrine therapy				
No	11 (68.8)	13 (68.4)	18 (78.3)	8 (80.0)
Yes	5 (31.3)	6 (31.6)	5 (21.7)	2 (20.0)

Abbreviations: DCO, data cutoff; ER+, estrogen receptor–positive; PgR+, progesterone receptor–positive.

### Safety

The occurrence of SAEs during the main study period and the follow-up period combined is detailed in Table 2. The majority of SAEs were considered by the investigator to be unrelated to the treatment. Two SAEs considered to be treatment related were documented (one case of hypertension and one case of pulmonary embolism, both in the fulvestrant 500 mg treatment group).

**Table 2.** Incidence of SAEs and Deaths

SAE	No. of Patients (%)	
	Fulvestrant 500 mg (n = 101)	Anastrozole 1 mg (n = 103)
Any SAE	24 (23.8)	22 (21.4)
Any SAE related to death	3 (3.0)	5 (4.9)
Any SAE with outcome other than death	21 (20.8)	18 (17.5)
Any causally related SAE	2 (2.0)	0
Most commonly reported (≥ two patients) SAEs		
Atrial fibrillation	1 (1.0)	1 (1.0)
Cardiac failure	2 (2.0)	0
Death	0	2 (1.9)
Decreased appetite	2 (2.0)	0
Dehydration	2 (2.0)	0
Dyspnea	2 (2.0)	0
Femur fracture	1 (1.0)	2 (1.9)
Neuralgia	1 (1.0)	1 (1.0)
Transient ischemic attack	0	2 (1.9)

Abbreviation: SAE, serious adverse event.

## DISCUSSION

This study reports improved OS with fulvestrant 500 mg treatment compared with anastrozole in the first-line setting for ER-positive advanced breast cancer, with an approximately 30% reduction in mortality risk. The previously reported improvements in TTP have translated into an improvement in OS of approximately 6 months with fulvestrant 500 mg (54.1 months) compared with anastrozole (48.4 months). This OS advantage is consistent with the OS benefit for fulvestrant 500 mg versus 250 mg in the second-line setting in the CONFIRM trial.^15^ The effect of fulvestrant 500 mg on OS was generally consistent across all prespecified subgroups (Fig 3). Furthermore, no new safety or tolerability issues were reported from the OS follow-up phase of this study, consistent with previously reported safety data.^16,17^

The improved OS with fulvestrant 500 mg (54.1 months) relative to anastrozole (48.4 months) was observed although the median OS for the anastrozole group in this study was higher than has previously been reported. For example, OS of 39.2 months was reported for anastrozole as first-line endocrine therapy for advanced breast cancer in a combined analysis of two phase III studies,^18^ and OS of 41.3 months was reported for the anastrozole monotherapy arm of a phase III combination study.^19^ In addition, corresponding median OS values of 34.0 months (letrozole)^20^ and 37.2 months (exemestane)^21^ have been reported for other AIs. It is therefore unlikely that the present analysis overestimates the margin of improvement with fulvestrant 500 mg over anastrozole, which might have been possible had the control arm underperformed.

The role of fulvestrant 500 mg as first-line therapy will be further defined by the ongoing phase III, double-blind FALCON (Fulvestrant and Anastrozole Compared in Hormonal Therapy Naïve Advanced Breast Cancer) trial (ClinicalTrials.gov identifier: NCT01602380). The FALCON trial will assess the efficacy of fulvestrant 500 mg versus anastrozole in women with locally advanced or metastatic breast cancer with strict definitions of endocrine therapy–naïve disease, including restrictions on exposure to hormone replacement therapy.

Endocrine therapy–naïve advanced breast cancer is relatively uncommon in countries with advanced health care, but represents a numerically substantial patient population, given the high disease prevalence. Furthermore, in unscreened populations and in developing countries, metastatic disease at presentation is a significant problem. Recent clinical trials reporting on first-line endocrine therapy in patients with ER-positive breast cancer have contained a substantial proportion, and often a majority, of endocrine therapy–naïve patients.^19,22–24^ In FIRST, previous endocrine therapy had been received by 29 (28.4%) of the patients treated with fulvestrant 500 mg and 23 (22.3%) of the anastrozole-treated patients. Of these 52 patients, only 3 had received AI previously (2 in the anastrozole group and 1 in the fulvestrant 500 mg group); the remainder had received adjuvant tamoxifen. Therefore, AI resistance resulting from previous AI exposure cannot account for the observed OS difference. Indeed, hypothetically, previous exposure to tamoxifen may bias against fulvestrant as both agents are in the same therapeutic class. Upon disease progression, patients were treated according to the standard of care, and therefore, there could potentially be imbalances between the two treatment groups that could have affected the OS analysis. However, response to subsequent therapies (systemic chemotherapy or endocrine therapy) has previously been shown to be similar between the treatment groups, demonstrating that patients with disease progression on fulvestrant retain sensitivity to subsequent treatments.^17^ Differential second-line response, therefore, is also an unlikely explanation for the observed OS effect.

There are significant limitations to this report. The sample size was relatively small, and the OS analysis was not specified in the original protocol but was added as a hypothesis in a protocol amendment after TTP results were known. Furthermore, 35 patients did not contribute additional data to the OS follow-up; the decision not to participate in the extended follow-up for OS was made solely by the patient or participating center and was known at the start of the OS follow-up and before the data were collected and analyzed. Data from these patients until the time of censoring were included in the OS analysis, and similar censoring patterns were seen in the two treatment groups. The sensitivity analyses support the main findings, that is, the differences in OS between treatment arms were unrelated to differences in censoring patterns. All-cause mortality was used to determine OS in this analysis because it is regarded as the most unbiased and objective end point used in oncology.^25^ This point is particularly relevant to an open-label study like FIRST. A final limitation was that the number of patients within subgroups was relatively small. Therefore, care should be taken when interpreting results.

Recent results from several trials with the cyclin-dependent kinase 4/6 (CDK4/6) inhibitor palbociclib are also pertinent to the discussion. PALOMA-1 (Palbociclib Ongoing Trials in the Management of Breast Cancer), a phase II trial of letrozole plus palbociclib versus letrozole alone, provided provisional US Food and Drug Administration approval for palbociclib in the first-line setting on the basis of PFS.^23^ No positive OS data have been reported to date; the results of a phase III trial of this comparison are pending (PALOMA-2, NCT01740427). Data from the phase III PALOMA-3 trial, comparing fulvestrant 500 mg plus palbociclib versus fulvestrant 500 mg alone in the second-line or subsequent setting in postmenopausal women (or pre- or perimenopausal women receiving goserelin), reported a marked PFS advantage for the combination, but OS data were also pending at the time of publication.^26^ The median PFS for fulvestrant 500 mg alone was shorter in PALOMA-3 than in previous studies, indicative of the younger, higher-risk, and more heavily pretreated population recruited into the PALOMA-3 trial.

The treatment algorithm for ER-positive advanced breast cancer, therefore, is in a state of flux. Currently, it is rational to consider fulvestrant 500 mg as a first-line treatment option given the potential for survival benefits, particularly in settings where palbociclib is not available or palbociclib cost or adverse effects are a significant concern, and especially if these results are confirmed in FALCON. These data also suggest that a first-line study of fulvestrant 500 mg with a CDK4/6 inhibitor versus fulvestrant 500 mg alone is a logical proposition that could lead to further prolonged TTP. Recent preclinical data on the efficacy of an ER degrading agent with a CDK4/6 inhibitor in ESR1-mutant breast cancer provides further rationale for this population, because improvements in TTP or OS could be caused by suppression of ESR1-mutant AI-resistant clones.^27^

In conclusion, we report that fulvestrant 500 mg may be associated with improved OS versus anastrozole in the first-line setting for ER-positive advanced breast cancer. To our knowledge, this represents the first time an endocrine monotherapy has demonstrated improved efficacy compared with a third-generation AI. The phase III FALCON trial may provide confirmation for these OS results; until then, the findings reported here should be regarded as preliminary, but clinically relevant.

## Supplementary Material

Protocol

## Glossary Terms

Anastrozole: a third-generation nonsteroidal aromatase inhibitor that prevents the conversion of androgen to estrogen in the peripheral tissues in postmenopausal women. Because hormone-dependent breast cancer progresses with estrogen, anastrozole has been used in the treatment of breast cancer in postmenopausal women. See *aromatase inhibitors*.
Aromatase inhibitors: inhibitors used in treating breast cancer in postmenopausal women. Aromatase inhibitors inhibit the conversion of androgens to estrogens by the enzyme aromatase, thus depriving the tumor of estrogenic signals. Because of decreased production of estrogen, estrogen receptors, which are important in the progression of breast cancer, cannot be activated.
Estrogen receptor (ER): ligand-activated nuclear proteins, belonging to the class of nuclear receptors, present in many breast cancer cells that are important in the progression of hormone-dependent cancers. After binding, the receptor-ligand complex activates gene transcription. There are two types of estrogen receptors (ERα and ERβ). ERα is one of the most important proteins controlling breast cancer function. ERβ is present in much lower levels in breast cancer, and its function is uncertain. Estrogen receptor status guides therapeutic decisions in breast cancer.
Overall survival: the duration between random assignment and death.
