# Supplementary material for: Fulvestrant 500 mg Versus Anastrozole 1 mg for the First-Line Treatment of Advanced Breast Cancer: Overall Survival Analysis From the Phase II FIRST Study
Source: J Clin Oncol. 2015 Sep 14;33(32):3781–7. doi: 10.1200/JCO.2015.61.5831 (PMC4737861; doi:10.1200/JCO.2015.61.5831)
Supplement: Protocol [file supp_33_32_3781__index.html]

Fulvestrant 500 mg Versus Anastrozole 1 mg for the First-Line Treatment of Advanced Breast Cancer: Overall Survival Analysis From the Phase II FIRST Study — Fulvestrant 500 mg: Overall Survival Versus Anastrozole — Protocol 

# Fulvestrant 500 mg Versus Anastrozole 1 mg for the First-Line Treatment of Advanced Breast Cancer: Overall Survival Analysis From the Phase II FIRST Study

## Protocol

The following protocol information is provided solely to describe how the authors conducted the research underlying this article. The information provided may not reflect the complete protocol or any previous amendments or modifications. As described in the Information for Contributors (http://jco.ascopubs.org/site/ifc/protocol.xhtml), *JCO* requests only specific elements of the most recent version of the protocol. The protocol information is not intended to replace good clinical judgment in selecting appropriate therapy and in determining drug doses, schedules, and dose modifications. The treating physician or other health care provider is responsible for determining the best treatment for the patient. ASCO and *JCO* assume no responsibility for any injury or damage to persons or property arising out of the use of this protocol material or due to any errors or omissions. Readers seeking additional information about the protocol are encouraged to consult the corresponding author directly.

Please click the protocol link below to access the information.

**Files in this Data Supplement:**

- Protocol
