# Supplementary material for: Fulvestrant 500 mg Versus Anastrozole 1 mg for the First-Line Treatment of Advanced Breast Cancer: Overall Survival Analysis From the Phase II FIRST Study
Source: J Clin Oncol. 2015 Sep 14;33(32):3781–7. doi: 10.1200/JCO.2015.61.5831 (PMC4737861; doi:10.1200/JCO.2015.61.5831)
Supplement: Protocol [file supp_JCO.2015.61.5831_Protocol_2015.615831.pdf]

The following protocol information is provided solely to describe how the authors conducted the research underlying the published report associated with the following article:

FULVESTRANT 500 MG VERSUS ANASTROZOLE 1 MG FOR THE FIRST-LINE TREATMENT OF ADVANCED BREAST CANCER: OVERALL SURVIVAL ANALYSIS FROM THE PHASE II 'FIRST' STUDY

Ellis, et al

DOI: 10.1200/JCO.2015.61.5831

The information provided may not reflect the complete protocol or any previous amendments or modifications. As described in the Information for Contributors (http://jco.ascopubs.org/site/ifc/protocol.xhtml) only specific elements of the most recent version of the protocol are requested by JCO. The protocol information is not intended to replace good clinical judgment in selecting appropriate therapy and in determining drug doses, schedules, and dose modifications. The treating physician or other health care provider is responsible for determining the best treatment for the patient. ASCO and JCO assume no responsibility for any injury or damage to persons or property arising out of the use of these protocol materials or due to any errors or omissions. Individuals seeking additional information about the protocol are encouraged to consult with the corresponding author directly.

**Revised Clinical Study Protocol**	
Drug Substance	ZD9238 (Fulvestrant)
Study Code	D6995C00006
Edition Number	5
Date	

A Randomised, Open-Label, Parallel-Group, Multi-centre, Phase II Study to Compare the Efficacy and Tolerability of Fulvestrant (FASLODEX™) 500 mg with Anastrozole (ARIMIDEX™) 1 mg as First Line Hormonal Treatment for Postmenopausal Women with Hormone Receptor Positive Advanced Breast Cancer

This submission /document contains trade secrets and confidential commercial information, disclosure of which is prohibited without providing advance notice to AstraZeneca and opportunity to object.

The following Amendment(s) and Administrative Changes are included in this amended protocol:

Amendment No.	Date of Amendment	Local Amendment No.	Date of local Amendment

Administrative change No.	Date of Administrative Change	Local Administrative change No.	Date of local Administrative Change

PROTOCOL SYNOPSIS

A Randomised, Open-Label, Parallel-Group, Multi-centre, Phase II Study to Compare the Efficacy and Tolerability of Fulvestrant (FASLODEX™) 500 mg with Anastrozole (ARIMIDEX™) 1 mg as First Line Hormonal Treatment for Postmenopausal Women with Hormone Receptor Positive Advanced Breast Cancer

International Coordinating Investigators

Study centre(s) and number of patients planned

A total of 200 patients will be recruited from approximately 50 centres in North America (10-30 patients), South America (10-50 patients) and Europe (120-180 patients). It is planned to recruit between 2 and 20 patients per centre, over an estimated period of 18 months.

Study period	Phase of development
Estimated date of first patient enrolled	II
Estimated date of last patient completed	

Objectives and Variables

Objectives	Variables
**Primary**	
To compare the clinical benefit rate (CBR) of patients treated with fulvestrant 500 mg with the clinical benefit rate of patients treated with anastrozole 1 mg.	Clinical Benefit (CB = CR + PR + SD ≥ 24 weeks defined by RECIST criteria)
**Secondary**	
**1.** To compare the objective response rate (ORR) of patients treated with fulvestrant 500 mg with the objective response rate of patients treated with anastrozole 1 mg	Objective Response (OR = CR + PR defined by RECIST criteria)
**2.** To compare the time to progression of patients treated with fulvestrant 500 mg with the time to progression of patients treated with anastrozole 1 mg	Time to Progression (TTP)
**3.** To describe the duration of response of patients treated with fulvestrant 500 mg and the duration of response of patients treated with anastrozole 1 mg.	Duration of Response (DoR)
**4.** To describe the duration of clinical benefit of patients treated with fulvestrant 500 mg and the duration of clinical benefit of patients treated with anastrozole 1 mg.	Duration of Clinical Benefit (DoCB)
**5.** To assess the safety and tolerability of fulvestrant 500 mg treatment compared with anastrozole 1 mg treatment.	Frequency and Severity of Adverse Events as assessed by CTC grade and laboratory assessments
**Exploratory**	
**1.** To explore the best overall response to the first subsequent systemic breast cancer therapy for patients randomised to fulvestrant 500 mg and patients randomised to anastrozole 1 mg.	Best overall response to subsequent therapy (CR, PR, SD ≥ 24 weeks, SD<24 weeks,PD or NE (not evaluable) as defined by the Investigator)

**2.** To evaluate subsequent clinical outcome in patients demonstrating changes in serum tumour markers for patients randomised to fulvestrant 500 mg and patients randomised to anastrozole 1 mg**.**	Serum tumour marker levels.

Study design

This is a randomised, open-label, parallel-group, multi-centre study.

Eligible patients will be randomised 1:1 to receive one of the following treatments:

- Fulvestrant 500 mg
- Anastrozole 1 mg

Target patient population

Postmenopausal women presenting with advanced breast cancer who have either never received endocrine therapy for advanced disease or have not received endocrine therapy in the preceding 12 months in the adjuvant setting**.**

Investigational product, dosage and mode of administration

Fulvestrant 500 mg will be administered as two 5 ml intramuscular injections, one in each buttock, on days 0, 14 (± 3), 28 (± 3) and every 28 (± 3 days) thereafter. Time windows extended to ± 7 days after 24 weeks.

Comparator, dosage and mode of administration

Anastrozole will be administered orally as a single daily tablet at a dose of 1 mg/day.

Duration of treatment

Treatment will continue until disease progression, unless any of the criteria for treatment discontinuation are met first.

Statistical methods

For CBR and ORR, the results will be expressed in terms of the odds ratio together with the corresponding 95% confidence interval (CI) and p-value. In addition, the estimate of the difference in CBR and ORR (fulvestrant – anastrozole) and the corresponding 2-sided 95% confidence interval will also be presented.

TTP will be analysed using a log-rank test (equivalent to the Cox's proportional hazards regression model with treatment factor only). The comparison of the treatment groups will be estimated using the hazard ratio of fulvestrant to anastrozole together with the corresponding 95% CI and p-value.

For TTP, DoR and DoCB, Kaplan-Meier plots and Kaplan-Meier estimates of the median time to event will be presented.

Safety and tolerability will be assessed in terms of laboratory data and frequency and severity of adverse events (AEs). AEs and laboratory data will be listed individually by patient and summarized according to treatment received.

The data cut-off for the primary analysis will be 6 months after the last patient is randomised. A further analysis of data collected during the follow-up phase will be performed when approximately 75% of patients are no longer receiving randomised study treatment.

At the DCO for the primary analysis, patients on fulvestrant 500 mg had a 60% longer TTP compared to patients on anastrozole 1 mg. The data maturity in terms of the proportion of patients with progression events was approximately 30%.

To investigate this finding further, a time to treatment failure (TTF) analysis and a more mature time to progression (TTP) analysis were planned for when 75% of patients had discontinued study treatment. For the follow-up analysis of TTP, progression will be defined by investigator opinion, as patients did not have formal RECIST visits in the follow-up period after the DCO for the primary analysis of the study.

In addition, an OS analysis will be performed for all randomised patients when approximately 133 (65%) of the patients have died.

TABLE OF CONTENTS PAGE

	TITLE PAGE	1
	PROTOCOL SYNOPSIS	3
	TABLE OF CONTENTS	7
	LIST OF ABBREVIATIONS AND DEFINITION OF TERMS	11
1.	INTRODUCTION	14
1.1	Background	14
1.2	Rationale for this study	15
1.2.1	Rationale for measurement of serum tumour markers	15
2.	STUDY OBJECTIVES	16
2.1	Primary objective	16
2.2	Secondary objectives	16
2.3	Exploratory objectives	17
3.	STUDY PLAN AND PROCEDURES	17
3.1	Overall study design and flow chart	17
3.2	Rationale and risk/benefit assessment	27
3.2.1	Rationale for study design, doses and control groups	27
3.2.1.1	Rationale for the dose of fulvestrant	27
3.2.1.2	Rationale for the choice of comparator	29
3.2.2	Risk/benefit and ethical assessment	29
3.3	Selection of study population	30
3.3.1	Study selection record	30
3.3.2	Inclusion criteria	30
3.3.3	Exclusion criteria	32
3.3.4	Restrictions	33
3.3.5	Discontinuation of patients from treatment or assessment	33
3.3.5.1	Criteria for discontinuation	33
3.3.5.2	Procedures for discontinuation	33
3.4	Treatments	34
3.4.1	Identity of investigational product and comparators	34
3.4.2	Doses and treatment regimens	34
3.4.3	Labelling	35
3.4.4	Storage	35
3.4.5	Accountability	35
3.5	Method of assigning patients to treatment groups	36
3.6	Blinding and procedures for unblinding the study (Not applicable)	37

3.7 Pre-study, concomitant and post-study treatment(s) ... 37
3.8 Treatment compliance ... 39
4. MEASUREMENTS OF STUDY VARIABLES AND DEFINITIONS OF OUTCOME VARIABLES ... 39
4.1 Primary variable ... 39
4.2 Screening and demographic measurements ... 39
4.3 Patient-Reported Outcomes (PROs) (Not applicable) ... 41
4.4 Health Economic measurements and variables (Not applicable) ... 41
4.5 Pharmacokinetic measurements and variables (Not applicable) ... 41
4.6 Efficacy and pharmacodynamic measurement and variables ... 41
4.6.1 Clinical benefit rate (CBR) ... 43
4.6.1.1 Methods of assessment ... 43
4.6.1.2 Derivation or calculation of outcome variable ... 43
4.6.2 Objective response rate (ORR) ... 43
4.6.2.1 Methods of assessment ... 43
4.6.2.2 Derivation or calculation of outcome variable ... 43
4.6.3 Time to Progression (TTP) ... 44
4.6.3.1 Methods of assessment ... 44
4.6.3.2 Derivation or calculation of outcome variable ... 45
4.6.4 Duration of response (DoR) ... 45
4.6.4.1 Methods of assessment ... 45
4.6.4.2 Derivation or calculation of outcome variable ... 45
4.6.5 Duration of Clinical Benefit (DoCB) ... 46
4.6.5.1 Methods of assessment ... 46
4.6.5.2 Derivation or calculation of outcome variable ... 46
4.6.6 Evaluation of serum-based disease tumour markers ... 46
4.6.6.1 Methods of assessment ... 46
4.6.6.2 Derivation or calculation of outcome variable ... 46
4.7 Safety measurements and variables ... 46
4.7.1 Adverse events ... 46
4.7.1.1 Definitions ... 46
4.7.1.2 Recording of adverse events ... 48
4.7.1.3 Reporting of serious adverse events ... 52
4.7.2 Laboratory safety measurements and variables ... 52
4.7.2.1 Methods of assessment ... 52
4.7.2.2 Derivation or calculation of outcome variables ... 53
4.7.3 Vital signs, ECG and physical examination ... 53
4.7.3.1 Methods of assessment ... 53
4.7.3.2 Derivation or calculation of outcome variables ... 54
4.8 Volume of blood sampling and handling of biological samples ... 54
4.8.1 Analysis of biological samples ... 55

5. DATA MANAGEMENT 55

6. STATISTICAL METHODS AND DETERMINATION OF SAMPLE SIZE 56

6.1 Statistical evaluation – general aspects 56

6.2 Description of outcome variables in relation to objectives and hypotheses 56

6.3 Description of analysis sets 57

6.4 Method of statistical analysis 57
6.4.1 Clinical benefit (CB) 57
6.4.2 Objective response (OR) 58
6.4.3 Time to progression (TTP) and Time to treatment failure (TTF) 58
6.4.4 Duration of response (DoR) 58
6.4.5 Duration of clinical benefit (DoCB) 58
6.4.6 Safety and tolerability 58
6.4.7 Exploratory analysis 59
6.4.8 Overall Survival 59

6.5 Determination of sample size 59

6.6 Interim analyses (Not applicable) 59

6.7 Data and safety monitoring board (Not applicable) 59

7. STUDY MANAGEMENT 59

7.1 Monitoring 59

7.2 Audits and inspections 60

7.3 Training of staff 60

7.4 Changes to the protocol 60

7.5 Study agreements 61

7.6 Study timetable and end of study 61

8. ETHICS 62

8.1 Ethics review 62

8.2 Ethical conduct of the study 62

8.3 Informed consent 62

8.4 Patient data protection 63

9. PROCEDURES IN CASE OF EMERGENCY, OVERDOSE OR PREGNANCY 64

9.1 AstraZeneca emergency contact procedure 64

9.2 Procedures in case of medical emergency 64

9.3 Procedures in case of overdose 64

9.4 Procedures in case of pregnancy 65
10. REFERENCES 65

LIST OF TABLES PAGE

Table 1 Study plan 22
Table 2 Study Plan (Follow-up phase) 25
Table 3 Ratio of animal/human exposure to fulvestrant on the basis of mean $AUC_{(0-28)}$ and C_{max} values following multiple doses 29
Table 4 Volume of blood to be drawn from each patient 55

LIST OF FIGURES PAGE

Figure 1 Study flow chart 21
Figure 2 Population-predicted profiles of fulvestrant 250 mg treatment and the planned 500 mg treatment regimen 28

LIST OF APPENDICES

Appendix A Signatures (not applicable)

Appendix B Additional Safety Information

Appendix C Objective Tumour Response Criteria (RECIST)

PROTOCOL SUPPLEMENTS

Supplement 1 Investigators and Study Administrative Structure

Supplement 2 Study Team Contacts in Event of Emergency Situations, Overdose or Pregnancy

LIST OF ABBREVIATIONS AND DEFINITION OF TERMS

The following abbreviations and special terms are used in this study protocol.

Abbreviation or special term	Explanation
AE	Adverse event (see definition in Section 4.7.1.1).
AP	Alkaline phosphatase
ALT	Alanine aminotransferase
AI	Aromatase inhibitor
ASA	Acetylsalicylic Acid (aspirin)
AST	Aspartate aminotransferase
Assessment	An observation made on a variable involving a subjective judgment
AUC	Area under the plasma concentration time curve from zero to infinity
BP	Blood pressure
CBR	Clinical benefit rate
CI	Confidence interval
C_{trough}	Trough concentration
C_{max}	Maximum concentration
CR	Complete response
CRA	Clinical Research Associate
CRF	Case Report Form
CRO	Contract Research Organisation
CT	Computerised tomography
d	day
DBL	Data Base Lock
DoCB	Duration of clinical benefit
DCIS	Ductal carcinoma in situ
DCO	Data Cut Off
DHEA	Dihydroepiandrosterone
DIC	Disseminated intravascular coagulation
DMC	Data Management Centre
DoR	Duration of response
ECG	Electrocardiogram

Abbreviation or special term	Explanation
Endpoint	A status of the patient that constitutes the 'endpoint' of a patient's participation in a clinical study and that is used as the final outcome.
ER	Oestrogen receptor
FDA	Food and Drug Administration
FSH	Follicle-stimulating hormone
GCP	Good Clinical Practice
HDPE	High density polyethylene
HR(on study plan only)	Heart rate
HR	Hormone receptor
HRT	Hormone replacement therapy
ICH	International Conference on Harmonisation
IEC	Independent Ethics Committee
im	Intramuscular
INR	International normalized ratio
IRB	Institutional Review Board
ITT	Intention-to-treat
L	Litre
LA	Long-acting
LD	Longest diameter
LH	Luteinising hormone
LH-RH	Luteinising hormone-releasing hormone
LMWH	Low Molecular Weight Heparin
LTFU	Lost to Follow Up
Measurement	An observation made on a variable using a measurement device.
MedDRA	Medical dictionary for Regulatory Activities
mg	Milligram
mL	Millilitre
monthly	28 ±3 days
MRI	Magnetic resonance imaging
NCI CTC	National Cancer Institute Common Terminology Criteria for Adverse Events
NCR	No carbon required

**Abbreviation or special term**	**Explanation**
NE	Not Evaluable
OAE	Other significant Adverse Event (ie, an adverse event of special interest in this clinical development; see definition in Section 4.7.1.1). AstraZeneca drug safety physicians will perform the classification of OAEs after the study is complete.
OR	Objective response
ORR	Objective response rate
OS	Overall Survival
pCRF	Paper Case Report Form
PD	Progressive disease
PFS	Pre-filled syringes
PgR	Progesterone receptor
po	By mouth
PK	Pharmacokinetics
PP	Per protocol
PR	Partial response
Principal Investigator	A person responsible for the conduct of a clinical study at an investigational study centre. Every investigational study centre has a principal investigator.
REC	Response Evaluation Committee
RECIST	Response Evaluation Criteria in Solid Tumours
SAE	Serious adverse event (see definition in Section 4.7.1.1).
SAP	Statistical analysis plan
SD	Stable disease
SERM	Selective Oestrogen Receptor Modulator
TTF	Time to Treatment Failure
TTP	Time to disease progression
ULRR	Upper limit of reference range
WHO	World Health Organisation

1. INTRODUCTION

Investigators should be familiar with the current fulvestrant Investigator's Brochure and the local prescribing information for anastrozole.

1.1 Background

Breast cancer is one of the most common female cancers and the most common cause of cancer deaths in women. It comprises 18% of all female cancers worldwide (McPherson et al, 2000). The incidence varies among populations with about half of all cases occurring in North America and Western Europe. Oestrogen acts as an endocrine growth factor for at least one third of breast cancers. It has long been acknowledged that many cancers are hormone dependent and that hormonal manipulation can affect the progress of the disease. The effects of hormonal manipulation on metastatic breast cancer were observed as early as 1896 following removal of the oestrogenic stimulus by bilateral oophorectomy (Beatson GT 1896). The most important factor determining response to hormonal manipulation is the presence of the oestrogen receptor in the target tissue (Fisher et al, 2001).

The anti-oestrogen tamoxifen (AstraZeneca ZD6157, NOLVADEX™) is the most widely used hormonal treatment for breast cancer in both pre- and postmenopausal women. This drug has been used to treat patients with breast cancer in advanced disease, as an adjuvant therapy after surgery, and for the treatment of ductal carcinoma in situ (DCIS), and also to reduce the risk of breast cancer development in women at high risk (Fisher et al, 1998). Despite its demonstrated efficacy in these patient populations, de novo resistance or acquired resistance may occur after prolonged treatment limiting the effectiveness of tamoxifen in many patients. In some patients the disease progresses during therapy because tumour growth may also be stimulated by tamoxifen (Weibe VJ et al, 1993).

Because the major source of oestrogen in postmenopausal women is aromatase-mediated conversion of circulating androstenedione to estrone in peripheral tissues, an alternative approach to management of breast cancer has been the use of aromatase inhibitors (AI). The development of oral, selective, non-steroidal AI led to the introduction of well-tolerated agents with clear evidence of clinical benefit. The third generation oral AIs, ie, AstraZeneca ZD1033 (anastrozole, ARIMIDEX™), letrozole (FEMARA™, Novartis, USA), and the steroidal, type I inhibitor, exemestane (AROMASIN™, Pfizer, USA), have now been tested in phase III trials where each has been shown to be more effective than the progestin megestrol acetate (MEGACE™, Bristol-Myers Squibb, USA) (Buzdar AU 1998, Buzdar et al, 2001, Dombernowsky et al, 1998, Kaufmann et al, 2000) and two have been shown to be more effective than tamoxifen (Bonneterre et al, 2001, Mouridsen et al, 2001).

Anastrozole greatly reduces estradiol levels in both serum and tumours in postmenopausal women leading to clinical benefits in women with hormone-responsive breast cancer (Geisler et al, 2001). Since its introduction, anastrozole has displaced older therapies (ie, progestins, androgens) and become widely used in patients whose disease has progressed on anti-oestrogen therapy (Buzdar AU 1998). Studies showing a survival advantage for patients

treated with anastrozole over the progestin megestrol acetate have made selective non-steroidal AIs the treatment of choice. At 31 months follow-up, anastrozole (1 mg) demonstrated a significant survival advantage over megestrol acetate (Buzdar AU 1998, Messori et al, 2000). AIs are now considered established first- and second-line hormonal agents.

1.2 Rationale for this study

Patients with advanced breast cancer will ultimately suffer progressive disease despite the efficacy of non-steroidal AIs. Therefore, therapies that provide longer disease control and clinical benefit are needed.

The search for an anti-oestrogen which is devoid of the agonist activity of tamoxifen and which can effectively block oestrogen receptor (ER) activity resulted in the discovery and clinical development of ZD9238 (fulvestrant, FASLODEX™). Fulvestrant is a new ER antagonist, without known agonistic properties, that downregulates cellular levels of the ER in a dose-dependent manner (Howell et al, 2000, Robertson JF et al, 2001, Wakeling AE et al, 1991). Fulvestrant is well-tolerated and has demonstrated efficacy in women whose breast cancer has progressed following tamoxifen therapy (Howell et al, 2002. Osborne CK et al, 2002). The results from two phase III trials showed that fulvestrant, given at a dose of 250 mg every 28 days, was at least as effective for both TTP and OR as anastrozole (1 mg/day).

This dose and schedule first received regulatory approval in the USA, and has since been approved in 34 markets worldwide, including Canada and the European Union. However, evidence from a number of studies suggest that higher doses of fulvestrant may be enhance efficacy further (see Section 3.2).

Study 9238IL/0025 demonstrated that fulvestrant 250 mg had similar efficacy to tamoxifen in the first line setting in the population of postmenopausal women with ER+ and/or PgR+ advanced breast cancer who had received no previous endocrine or cytotoxic therapy (Robertson JF et al, 2002). The treatment population in Study 25, however, included a significant proportion of patients with hormone receptor negative and unknown status which may have contributed to the inability to meet the primary endpoint of the trial in the intention-to-treat population. In addition, increased knowledge of fulvestrant has led to the hypothesis that the currently approved dosing regimen for fulvestrant while effective, may not be optimal. The current study is therefore designed to explore the hypothesis that an optimised dosing regimen for fulvestrant that includes both a loading dose component to reach steady state faster and a higher dose to achieve overall higher exposure to fulvestrant may be a better first line therapy option than the current standard, anastrozole.

1.2.1 Rationale for measurement of serum tumour markers

The measurement of circulating tumour markers may offer an effective and objective method of monitoring disease remission and progression in patients receiving a systemic therapy for metastatic breast cancer. Serum tumour markers are easily obtained and can be measured serially as a surrogate endpoint reflecting the dynamic changes in tumour burden. Assessment

of a marker and the correlative therapeutic outcome may be important for better targeting and monitoring of therapy in the management of these patients.

The clinical interpretation of serum tumour markers is not simple. Evidence suggests that markers may rise in the first few months of treatment without this necessarily indicating an imminent progression in disease status. This phenomenon may lead to some investigators inappropriately terminating treatment early.

Serum samples will be collected in this study and evaluated for a panel of existing tumour markers (to possibly include, but not be limited to CA15-3, CA27.29, ECD-HER-2/neu and CEA), as well as markers yet to be determined in order to assess their prognostic and/or predictive value of treatment response and clinical outcome.

2. STUDY OBJECTIVES

2.1 Primary objective

**Primary Objective**	**Variable**
To compare the clinical benefit rate of patients treated with fulvestrant 500 mg with the clinical benefit rate of patients treated with anastrozole 1 mg.	Clinical Benefit (CB = CR + PR + SD ≥ 24 weeks defined by RECIST criteria)

2.2 Secondary objectives

**Secondary Objectives**	**Variables**
**1.** To compare the objective response rate of patients treated with fulvestrant 500 mg with the objective response rate of patients treated with anastrozole 1 mg	Objective Response (OR = CR + PR defined by RECIST criteria)
**2.** To compare the time to progression of patients treated with fulvestrant 500 mg with the time to progression of patients treated with anastrozole 1 mg	Time to Progression (TTP)
**3.** To describe the duration of response of patients treated with fulvestrant 500 mg and the duration of response of patients treated with anastrozole 1 mg.	Duration of Response (DoR)

Secondary Objectives	Variables
**4.** To describe the duration of clinical benefit of patients treated with fulvestrant 500 mg and the duration of clinical benefit of patients treated with anastrozole 1 mg.	Duration of Clinical Benefit (DoCB)
	Frequency and Severity of Adverse Events as assessed by CTC grade and laboratory assessments

2.3 Exploratory objectives

Exploratory Objectives	Variable
**1.** To explore the best overall response to the first subsequent systemic breast cancer therapy for patients randomised to fulvestrant 500 mg and patients randomised to anastrozole 1 mg	Best overall response to subsequent therapy (CR, PR, SD $\geq$ 24 weeks, SD<24 weeks, PD or NE as defined by the Investigator)
**2.** To evaluate subsequent clinical outcome in patients demonstrating changes in serum tumour markers for patients randomised to fulvestrant 500 mg and patients randomised to anastrozole 1 mg.	Serum tumour marker levels.

3. STUDY PLAN AND PROCEDURES

3.1 Overall study design and flow chart

This Clinical Study Protocol has been subjected to a peer review according to AstraZeneca standard procedures.

This is a randomised, open-label, parallel-group, multi-centre study.

Target patient population:

200 postmenopausal women presenting with advanced breast cancer who have either never received endocrine therapy for advanced disease or have not received endocrine therapy in the preceding 12 months in the adjuvant setting.

The study will be open to patients with measurable disease as per RECIST criteria and/or bone lesions (lytic or mixed [lytic + sclerotic]). All randomised patients will be included in the analysis of clinical benefit – patients with no measurable disease may qualify for clinical benefit by remaining progression-free for at least 24 weeks. However, the analysis of the secondary endpoint of objective response will be based only on those patients with measurable disease. Tumour markers will not be used to assess efficacy.

Treatment Schedule:

Patients who meet the eligibility criteria will be randomised 1:1 into one of the following treatment groups:

1. Fulvestrant 500 mg im on days 0, 14 (± 3), 28 (± 3) and every 28 (± 3 days) thereafter. Time windows extended to ± 7 days after 24 weeks.

2. Anastrozole 1 mg po once daily.

Treatment will continue until disease progression as defined by the protocol, unless any of the criteria for treatment discontinuation were met first.

At least one set of films/electronic images must be made at each tumour assessment and stored with the hospital records. Copies of the films/electronic images will be sent at regular intervals to the REC (Response Evaluation Committee) for independent review. An independent panel (REC) consisting of radiologists/oncologists will perform a review of patients with a complete response (CR), partial response (PR) or stable disease (SD) ≥ 24 weeks. An additional review of patients with progressive disease (PD) may be undertaken to explore any disparity between known progression rates for hormonal therapy in the first line setting and those emerging in this trial. The reviewers will be blinded to treatment but not to the sequence of clinical visits. The REC's decision will be used to corroborate the analysis from the investigators assessment. The investigators assessment will be used for the primary analysis. The primary analysis will be based on RECIST information, which will be programmatically derived.

The data cut-off for the primary analysis will be 6 months after the last patient has been recruited. Prior to this date, all patients must be followed up for disease progression every 12 weeks as defined by the protocol (see Study Plan [Table 1]), regardless of whether they are still receiving randomised treatment.

Follow up phase

Upon disease progression as defined by the protocol, patients will enter the follow-up phase and be treated as per standard clinical practice (see Study Plan – Follow-up phase [Table 2]).

After the data cut-off for the primary analysis, all the remaining patients, regardless of whether they are still receiving randomised treatment, will enter the follow-up phase and be followed as per standard clinical practice (see Study Plan – Follow-up phase [Table 2]).

A follow-up questionnaire will be completed for all patients 12 months after entering the follow-up phase. Thereafter, follow-up questionnaires will only be completed every 12 months for patients that continue to receive randomised treatment, until a further analysis of TTF and TTP analysis is performed.

The further analysis of data collected during the follow-up phase will be performed when approximately 75% of patients have discontinued randomised study treatment. The endpoints which will be analysed are TTF and TTP.

At the DCO for the primary analysis, patients on fulvestrant 500 mg had a 60% longer TTP compared to patients on anastrozole 1 mg. The data maturity in terms of the proportion of patients with progression events was approximately 30%.

To investigate this finding further, a time to treatment failure (TTF) analysis and a more mature time to progression (TTP) analysis were planned for when 75% of patients had discontinued study treatment. For the follow-up analysis of TTP, progression will be defined by investigator opinion, as patients did not have formal RECIST visits in the follow-up period after the DCO for the primary analysis of the study.

Randomisation will be stratified by centre and the randomisation schemes will be provided by the Biostatistics Department of AstraZeneca. Patients will be allocated to randomised treatment by investigators strictly sequentially (see Section 3.5).

Overall Survival

An OS analysis will be performed for all randomised patients when approximately 133 (65%) of the patients have died.

The patients' survival status will be recorded approximately every 3 months until one or more of the following occurs: -

- The patient is known to have died
- The patient had previously withdrawn consent to participate in the study.
- The patient is deemed permanently lost to follow up*
- The study analysis time point is reached, and this information is no longer required.
- Additional Informed Consent** is not given, or that consent is withdrawn

* If the patient is deemed permanently lost to follow up, the date the patient was last known to be alive will, where possible, be recorded.

** Additional informed consent is required for the collection of survival data for all subjects who are still alive.

Patients still on study and receiving study drug

OR

Patients previously on study, still attending the Investigator site clinic for routine cancer care, but no longer receiving study drug

- The Investigator will explain to the patient that a further statistical analysis for the study is planned. It will be emphasised that this will entail only the collection of survival information, and will not involve any new study procedures, nor will it change the breast cancer care they receive.

- Patients will be asked to sign an additional consent form to say they understand this and give their permission to collect this data.

- Patients previously on study but who are now deceased, or who no longer attend the Investigator site clinic for routine cancer care

- If the patient's death has already been reported, and all necessary information is recorded on the database, then no further action is required.

- If the patient's death occurred whilst still on study, but the necessary information was not recorded on the database (the collection of death was not routinely done in the follow-up stage of the study), then the missing information will be requested from the Investigator/patient notes and entered onto the study database.

- If the patient is known to have died, but the death did not occur whilst the patient was still being followed under the study protocol, then, conditional on ethical committee approval, this information will be requested from the Investigator/patient notes and entered onto the study database.

- If the patient's survival status is not known, and the necessary ethical and regulatory approvals have been granted, then every effort should be made to obtain the patient's survival status from other sources e.g. a new cancer care provider, patients general medical practitioner. If applicable, site staff could utilise a death registry that is in the public domain, to obtain information.

- In this scenario, if the subject is alive, then a signed additional informed consent form is required. If the patient is alive and declines to provide the additional consent, then no survival data can be collected.

- If the patient is deemed permanently lost to follow up, the date the patient was last known to be alive will be recorded when possible.

Figure 1 Study flow chart

200 postmenopausal women with hormone receptor positive advanced breast cancer

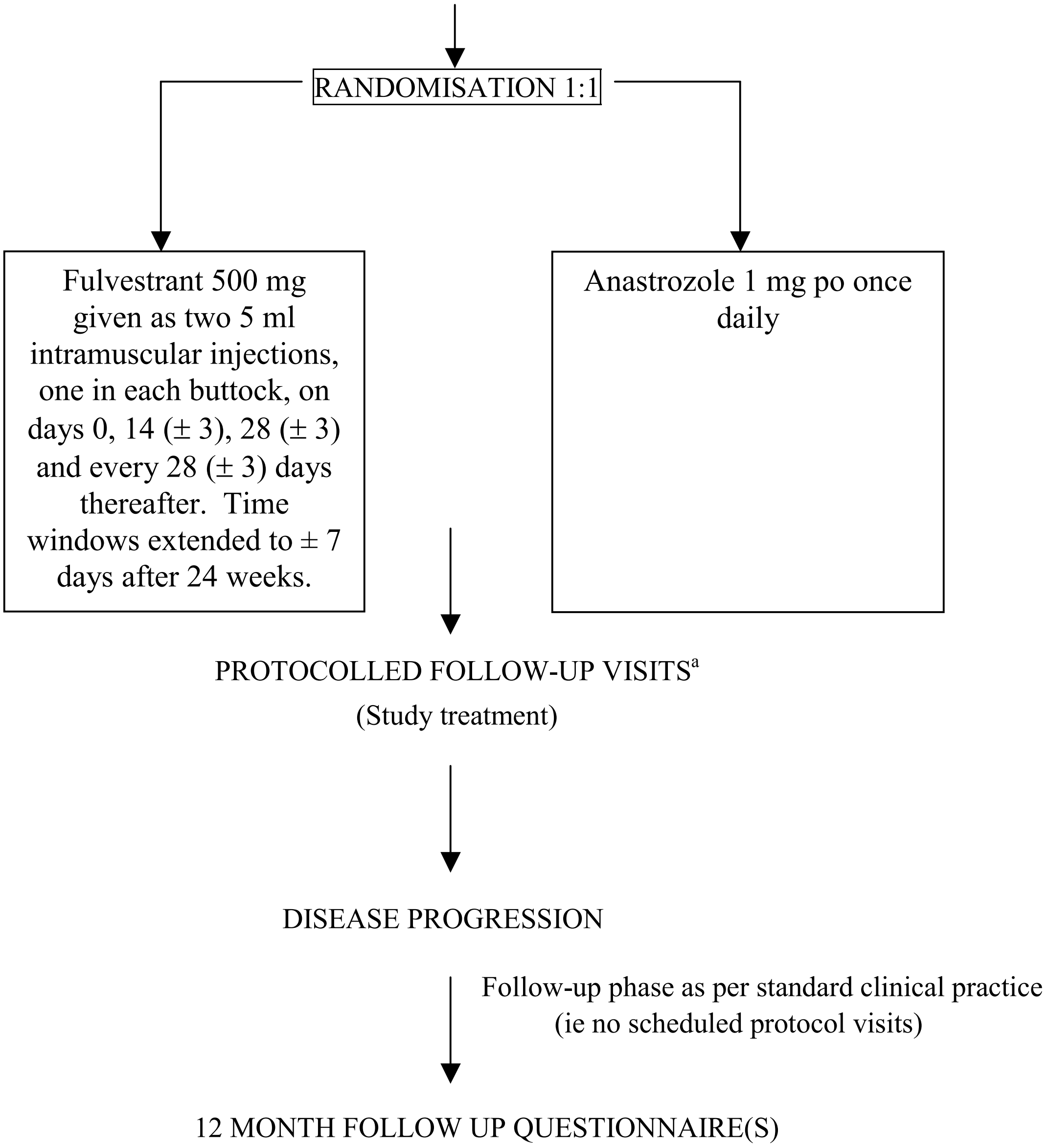

[a] The data cut-off for the primary analysis will be 6 months after the last patient has been recruited. Prior to this date, all patients must be followed up for disease progression as defined by the protocol (see Study Plan [Table 1]), regardless of whether they are still receiving randomised treatment.

Upon disease progression as defined by the protocol, patients will enter the follow-up phase and be treated as per standard clinical practice (see Study Plan – Follow-up phase [Table 2]).

After the data cut-off for the primary analysis, all the remaining patients will enter the follow-up phase. An analysis of data collected during the follow-up phase will be performed when approximately 75% of patients have discontinued randomised study treatment. The endpoints which will be analysed are TTF and TTP. An OS analysis will be performed for all randomised patients when approximately 133 (65%) of patients have died

Table 1 Study plan

Study plan	Screening Phase	Treatment Phase									Treatment Discontinuation [n]
Visit	Screening[a]	1 [b]	2 [k] (Day 14)	3	4	5	6	7	8	9[+] onwards (every 12 weeks until progression)	
Week(s)	-3 to 0	0	2 (± 3d)	4 (± 3d)	8 (± 3d)	12 (± 3d)	16 (± 3d)	20 (± 3d)	24 (± 3d)	36 (± 7d) and Onwards	
Informed consent	X										
Medical history	X										
Demography	X										
Inclusion/exclusion criteria	X										
Concomitant therapy	X	X		X	X	X	X	X	X	X	X [c]
ECG [d]	X [d]										
Physical Examination	X	X			X		X		X	X	X
WHO Performance Status	X	X			X		X		X	X	X
Vital Signs (BP, HR)		X			X		X		X	X	X
Height		X									
Weight		X		X	X	X	X	X	X	X	X
Haematology/Biochemistry [e]	X	X		X		X			X	X	X
Serum-based tumour markers		X		X		X			X		X
Tumour assessment [f] (clinical and radiological)	X [g]					X			X	X	X
Chest X-ray, CT or MRI scan [f, h, i]	X [g]					(X)			(X)	(X)	(X)
Abdominal CT or MRI scan [f, h]	X [g]					(X)			(X)	(X)	(X)
Bone scan or skeletal survey	X [j]								(X) [j]	(X) [j]	(X) [j]

Table 1 Study plan

Study plan	Screening Phase	Treatment Phase									Treatment Discontinuation [n]
Visit	Screening[a]	1 [b]	2 [k] (Day 14)	3	4	5	6	7	8	9+ onwards (every 12 weeks until progression)	
Week(s)	-3 to 0	0	2 (± 3d)	4 (± 3d)	8 (± 3d)	12 (± 3d)	16 (± 3d)	20 (± 3d)	24 (± 3d)	36 (± 7d) and Onwards	
Skeletal X-rays, CT or MRI scan	(X) [j]					(X) [j]			(X) [j]	(X) [j]	(X) [j]
Randomised Treatment		X	X [k]	X	X	X	X	X	X [l]	X [l]	
Adverse events [m]	X	X		X	X	X	X	X	X	X	X [c]

() Assessments shown in brackets eg (x) are only mandatory if the baseline assessment showed evidence of metastatic lesions. Additional assessments may be performed if clinically indicated.
a Within 3 weeks before randomisation.
b Visit 1/Day 0 should occur no more than 1 week after randomisation and no more than 4 weeks after tumour assessment.
c Adverse event (AE) and concomitant therapy follow-up - 8 weeks after last injection of fulvestrant or 30 days after the last anastrozole tablet. First subsequent systemic breast cancer therapy received following discontinuation of randomised treatment and details of response to treatment will also be collected.
d An electrocardiogram (ECG) assessment should be recorded within 3 weeks prior to randomisation and repeated should any cardiac adverse events occur.
e Laboratory assessments (haematology and biochemistry) will be performed before randomisation, before treatment (unless treatment is given within 7 days following screening assessments), at weeks 4 and 12, and every 12 weeks thereafter, until withdrawal from randomised treatment.
f Assessment by RECIST Criteria every 12 (± 2) weeks from Visit 1 (not from screening date) until progression. Tumours or lesions evident on screening evaluations will be followed using same methodology at each assessment. For patients with an objective response of CR or PR, confirmations of response by repeat imaging must be performed at 4 weeks (or as soon as possible thereafter) following the date of response. Appropriate evaluation of new signs or symptoms suggestive of metastatic disease should be conducted as required to evaluate progression.
g Within the 4 weeks before treatment.
h CT and MRI are the best currently available and most reproducible methods for evaluating target lesions selected for response assessment. Conventional CT and MRI should be performed with contiguous cuts of 10 mm or less in slice thickness. Spiral CT should be performed by use of a 5 mm contiguous reconstruction algorithm.
i It is recommended that lesions identified by X-ray are confirmed by CT or MRI scan and that CT or MRI scanning be used to follow the lesions at each subsequent tumour assessment.
j Patients must have an isotopic bone scan or a skeletal survey (limited to X-ray films of skull (AP and lateral), total spine (AP and lateral), clavicle, ribs, pelvis, upper humeri and femori) within 8 weeks before treatment. Any hotspots identified on the bone scan must be confirmed by X-ray, computerised tomography (CT) scan or magnetic resonance imaging (MRI) within 4 weeks prior to treatment (a skeletal survey done within 4 weeks of treatment does not require further confirmation) and followed up every 12 (± 2) weeks using the same methodology for comparison with the baseline scans. All patients with metastatic bone lesions at baseline, must have isotopic bone scans or skeletal surveys every 24 (± 2) weeks until progression. Additional bone scans or skeletal surveys should be performed if clinically indicated. All abnormalities found on subsequent bone scans must also be confirmed by X-ray, CT scan, or MRI and followed up every 12 (± 2) weeks using the same methodology for comparison with the screening studies.
k Only patients randomised to fulvestrant 500 mg need to attend this visit
l Treatment continues to be given/dispensed every 28 (± 7) days after week 24.
m Both SAEs and AEs are collected during this period

n Prior to the data cut-off date for the primary analysis:

- Patients who discontinue randomised treatment for any other reasons than disease progression and withdrawal of consent will continue to be followed up for progression as defined by the protocol every 12 weeks.
- Upon disease progression as defined by the protocol, patients will enter the follow-up phase of the study and be treated as per standard clinical practice (see Study Plan – Follow-up phase (Table 2))
- After the data cut-off for the primary analysis, all the remaining patients will enter the follow-up phase (see Study Plan – Follow-up phase (Table 2)). A further analysis of data collected during the follow-up phase will be performed when approximately 75% of patients have discontinued randomised study treatment. The endpoints which will be analysed are TTF and TTP. An OS analysis will be performed for all randomised patients when approximately 133 (65%) of patients have died.

Upon disease progression as defined by the protocol, patients will enter the follow-up phase and be treated as per standard clinical practice. After the data cut-off date for the primary analysis, all the remaining patients, regardless of whether they are still receiving randomised treatment, will enter the follow-up phase and be followed as per standard clinical practice.

Table 2 Study Plan (Follow-up phase)

Study plan	Follow-Up Phase (as per standard clinical practice)[a]
Visit	12 month follow-up questionnaire(s)
Week(s)	52 weeks follow-up questionnaire(s) - +/- 3 weeks
**Pts receiving randomised treatment (after the data cut-off for the primary analysis)**	
WHO Performance Status	X
Serious Adverse Events[b]	X
Randomised treatment[c]	X
Progression status[d]	X
Subsequent breast cancer therapy details	X
Best response to 1st subsequent breast cancer therapy[e]	X
**Patients who have discontinued randomised treatment (at or before the data cut-off for the primary analysis)**	
Progression status (if applicable)[d]	X
Subsequent breast cancer therapy details	X
Best response to 1st subsequent breast cancer therapy[e]	X
**OS Survival Data Collection for all randomised patients for which death has not been previously recorded**	
Additional patient consent[f]	X
Survival Status (collected approximately 3 monthly)	X
Statement of Death (if applicable)	X

[a] All patients entering the follow-up phase will be treated as per standard clinical practice (ie there will be no scheduled protocol visits). A follow-up questionnaire will be completed for all patients 12 months after entering the follow-up phase. Thereafter, follow-up questionnaires will only be completed every 12 months for patients that continue to receive randomised treatment until the DCO for the TTF/TTP data analysis is reached

[b] During the follow up phase, SAEs need only be reported for patients still receiving randomised treatment. SAEs for these patients should be collected for up to 8 weeks after the last injection of fulvestrant, or 30 days after the last anastrozole tablet is taken.

[c] Randomised treatment may continue to be given/dispensed for as long as the patient receives clinical benefit.

[d] Details of whether the patient's disease has progressed, as assessed by the Investigator, will be collected.

e Details of the best overall response (CR, PR, SD, PD or non-evaluable as defined by the Investigator) and duration of stable disease (> 24 weeks or < 24 weeks), to the first subsequent systemic breast cancer therapy and will be collected until the final DCO occurs for the TTF/TTP data analysis.

[f] All randomised patients will be asked to sign an additional consent form for the collection of additional OS data, except those who are dead, Lost to Follow Up (LTFU) or have previously withdrawn consent to participate in the study.

3.2 Rationale and risk/benefit assessment

This is a phase II randomised, open-label, parallel-group, multi-centre study to compare fulvestrant (500 mg every 28 days plus an additional 500 mg on day 14 of the first month only) with anastrozole (1 mg/day) in the treatment of first line advanced breast cancer.

3.2.1 Rationale for study design, doses and control groups

3.2.1.1 Rationale for the dose of fulvestrant

Despite advances in the treatment of postmenopausal women with hormone receptor positive advanced breast cancer, almost all patients will ultimately undergo disease progression. Therefore the development of more effective hormonal strategies is needed. Fulvestrant at a dose of 250 mg every 28 days is the first oestrogen receptor antagonist shown to be at least as effective for both TTP and OR as a third-generation aromatase inhibitor in the second-line treatment of advanced breast cancer (Howell et al, 2002, Osborne CK et al, 2002). In these studies, overall survival was also similar between the fulvestrant and anastrozole treatment arms (Pippen J et al, 2003). However, evidence from a number of studies suggests that a higher dose may be able to enhance efficacy further.

The hypothesis that greater efficacy may be achieved using an increase in fulvestrant dose is based on:

- Data from studies 9238IL/0020 (Howell et al, 2002) and 9238IL/0021 (Osborne CK et al, 2002) suggesting that a dose-response effect exists for fulvestrant. These studies also included a lower dose arm (125 mg fulvestrant every 28 days) which was discontinued because it failed to meet the minimum efficacy requirements.

- Data from study 9238IL/0036 (Addo et al 2002) also suggest that a dose-response relationship may exist. In female volunteers given a single im injection of fulvestrant, there was a dose-dependent inhibition of ethinyloestradiol-induced endometrial thickening seen at day 28.

- Results from short term exposure to fulvestrant in Studies 9238IL/0002 (DeFriend et al, 1994) and 9238IL/0018 (Robertson JF et al, 2001) showing that expression of ER, PgR and the cell proliferation-related antigen Ki67 are reduced in a dose dependent manner and suggesting that the maximum effect has not been reached.

- The pharmacokinetic (PK) modelling (see Figure 2) providing evidence that an increase in fulvestrant dose and dosing schedule will result in a:

  decreased time to steady state plasma levels

  higher AUC and C_{max}

  higher trough level and therefore maintenance of a higher exposure throughout the dosing interval

At present, no dose-ranging trials to assess efficacy beyond the 250 mg every 28 days dosing schedule have been completed. The delivery of a higher dose of fulvestrant in a 5 ml volume is currently constrained by the limits of the formulation, and what is perceived as being a clinically acceptable schedule of administration. However, because of the favourable safety profile of fulvestrant, administration of a higher dose (volume) may be acceptable if it is paired with greater efficacy. The delivery of a higher dose, as predicted by pharmacologic modelling, may lead to the rapid achievement of steady state (and maintenance of C_{trough}).

In this study, fulvestrant will be administered at a dose of 500 mg every 28 days plus an additional 500 mg dose on day 14 of the first month only in an attempt to decrease the time to achieve steady-state levels and increase efficacy. Phase III pharmacokinetic (PK) sampling data has demonstrated that a 250 mg dose administered every 28 days requires 3 to 6 months (90 to 180 days) before approaching steady-state drug concentrations. Earlier achievement of steady-state plasma concentrations may be important to avoid earlier "progression events".

Figure 2 Population-predicted profiles of fulvestrant 250 mg treatment and the planned 500 mg treatment regimen

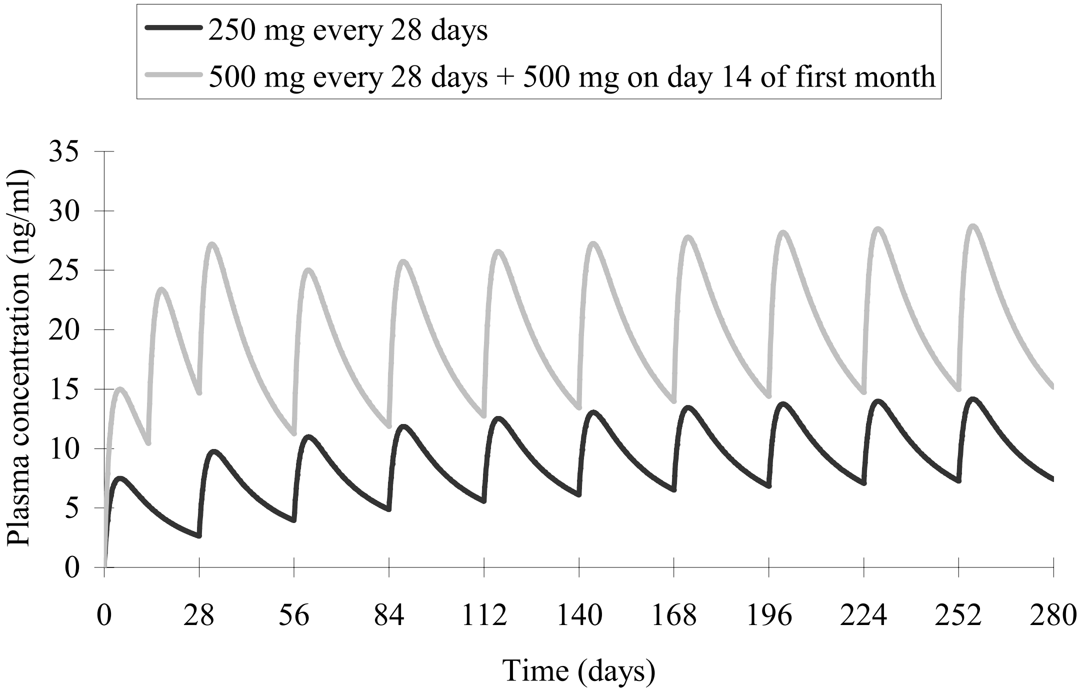

The expected mean peak plasma concentration (C_{max}) is approximately 27 ng/mL, around day 32. Over the next 10 months, C_{max} will be expected in the range of 26 – 28 ng/mL. The trough plasma concentrations at steady state are expected to be approximately 15 ng/mL compared to approximately 7 ng/mL for the 250 mg every 28 days injection.

3.2.1.2 Rationale for the choice of comparator

As mentioned in Section 1.1, anastrozole at a dose of 1 mg/day is an established treatment for first line advanced breast cancer.

3.2.2 Risk/benefit and ethical assessment

The predicted exposure during the first month of dosing the 500 mg fulvestrant regimen is 1.5 fold higher than the steady state exposure (approximately 10,800 ng·h/mL: vs 7245ng·h/mL) achieved with the standard every 28 day administration of the 250 mg intramuscular dose (See Table 3). In previous trials, some patients were treated on the standard 250 mg every 28 day schedule for more than 10 months achieving a high total exposure over the course of the study. This did not result in any worsening of the adverse event profile over time. In addition, fulvestrant has previously been given at a dose of 10 mg intravenously over 1 hour to healthy subjects (Study 9238IL/0026). The mean peak plasma concentration after intravenous (iv) administration was 140ng.ml^{-1}, with 1 subject having a peak drug level of 260 ng.ml^{-1} at the end of 1 hour. Plasma levels of fulvestrant were above 30 ng.ml^{-1} for about 1.5 hours and subjects were monitored for an additional 48 hours after the dose with no significant adverse reactions reported.

Table 3 Ratio of animal/human exposure to fulvestrant on the basis of mean $AUC_{(0-28)}$ and C_{max} values following multiple doses

Parameter	Rat (male)	Rat (female)	Dog	Human[a]	Human[b] (predicted)
Dose	10 mg/rat/15 days	10 mg/rat/15 days	40 mg/kg/28 days	250 mg/28 days	500 mg/28 days
AUC ng.h/ml	46,656	92,688	36000	7245	10800
AUC Ratio [c]	4.3	8.6	3.3	0.7	1.0
C_{max} ng/ml	105	372	88	14.6	28
C_{max} Ratio [c]	3.8	13.3	3.1	0.5	1.0

a Studies 0021 and 0020 (steady-state parameter estimates).
b Based on proposed high dose regimen (500 mg) in this study (parameters over the 1st four weeks).
c Relative to predicted human exposure in the high dose regimen (500 mg)

In addition, when adverse event data was compared against pre-clinical toxicology information (Table 3), no issues seemed evident.

This information, together with the estimated low inter-patient variability of 28%, suggest that the exposure predicted for the 500 mg fulvestrant dose regimen in terms of the anticipated C_{max} and AUC is not expected to cause major safety concerns.

Patients randomised to anastrozole 1 mg daily are being randomised a standard of care that is at least as good as that which they would otherwise have been exposed to had they not entered the study, but they will benefit from more frequent and detailed assessments.

Patients randomised to 500 mg fulvestrant will be receiving twice the strength of the currently marketed dose (plus an additional 500 mg dose on day 14). The currently-marketed dose of fulvestrant has been shown in clinical trials (Howell et al, 2002) to have efficacy and tolerability that is at least comparable to that of anastrozole which is itself currently the perceived optimum standard of care in many countries. The additional risks and benefits that may be attributed to this trial therefore reside in the additional amount of drug that patients will be receiving over and above the marketed dose in the open label fulvestrant arm. It is believed that the potential gains in benefit will outweigh any, as yet undefined, risks associated with dose-related toxicities.

The open-label nature of this trial means that the investigators will not be blinded to those patients being exposed to additional fulvestrant and it is therefore envisaged that any adverse events that do occur with these patients will be identified earlier than would have been the case if the trial had been a double blind double dummy trial.

The open-label nature of the trial has been chosen because the mode of administration of test compound involves intra-muscular gluteal injection and investigators and patients have both reported that this kind of imposition with placebo formulation would not be a favourable consideration. Previously this additional benefit (to those patients who avoid placebo injections) has been considered an acceptable risk (of being open label) by regulatory authorities, ethics committees and institutional review boards alike.

3.3 Selection of study population

3.3.1 Study selection record

Before entering the study, patients will be assessed to ensure that they meet the eligibility criteria. Patients not meeting these criteria should not be entered into the study. The investigator must keep a record of patients who were considered for enrolment but were never enrolled, eg, patient screening log indicating the reason why they were not enrolled. This information is necessary to establish that the patient population was selected without bias. The patient-screening log should be filed in the Investigator Study File at each centre.

Note: The patient's signed and dated informed written consent must be obtained before conducting any procedure specifically for the study.

3.3.2 Inclusion criteria

For inclusion in the study, patients must fulfil all of the following criteria:

1. Provision of written informed consent

2. Histological/cytological confirmation of breast cancer

3. Documented positive hormone receptor status (ER +ve and/or PgR + ve) of primary or metastatic tumour tissue, according to the local laboratory parameters

4. Patients with metastatic or locally advanced disease not amenable to therapy with curative intent:

   who have never had hormonal therapy for loco regionally advanced or metastatic disease

   and

   For patients who have received previous adjuvant or neoadjuvant hormonal treatment, this must have been completed more than 12 months prior to randomisation.

   Note: Adjuvant and neoadjuvant treatment may have included more than one hormonal agent.

5. Patients fulfilling one of the following criteria:

- Patients with measurable disease as per RECIST criteria. This is defined as at least one lesion that can be accurately measured in at least one dimension (longest diameter to be recorded) as ≥ 20 mm with conventional techniques or as ≥ 10 mm with spiral CT scan. Previously irradiated lesions will not be considered measurable unless there is a definite progression at such lesions at the screening tumour assessments.
- Patients with at least one bone lesions, lytic or mixed (lytic + sclerotic), in the absence of measurable disease as defined by RECIST criteria, which has not been previously irradiated

6. Postmenopausal woman, defined as a woman fulfilling any 1 of the following criteria:

- Age ≥ 60 years
- Age ≥ 45 years with amenorrhoea ≥ 12 months with an intact uterus
- Having undergone a bilateral oophorectomy
- FSH and oestradiol levels in postmenopausal range (utilising ranges from the local laboratory facility)*

  * In patients who have previously been treated with an LH-RH analogue, the last depot must have been administered more than 4 months prior to randomisation and menses must not have restarted.

7. WHO performance status 0, 1 or 2.

3.3.3 Exclusion criteria

Any of the following is regarded as a criterion for exclusion from the study:

1. Presence of life-threatening metastatic visceral disease, defined as extensive hepatic involvement, or any degree of brain or leptomeningeal involvement (past or present), or symptomatic pulmonary lymphangitic spread. Patients with discrete pulmonary parenchymal metastases are eligible, provided their respiratory function is not significantly compromised as a result of disease

2. Previous systemic therapy for advanced breast cancer.

3. Treatment with a non-approved or experimental drug within 4 weeks before randomisation

4. Current or prior malignancy within previous 3 years (other than breast cancer or adequately treated basal cell or squamous cell carcinoma of the skin or in-situ carcinoma of the cervix)

5. Any of the following laboratory values within 3 weeks of randomisation:

- Platelets < 100×10^9 / L
- Total bilirubin > 1.5 × ULRR**

  ** Patients with confirmed Gilbert's syndrome may be included in the study

- ALT or AST > 2.5 × ULRR if no demonstrable liver metastases or > 5 × ULRR in presence of liver metastases

6. History of :

- bleeding diathesis (ie, disseminated intravascular coagulation [DIC], clotting factor deficiency) or
- long-term anticoagulant therapy (other than antiplatelet therapy and low dose warfarin – see Section 3.7)

7. History of hypersensitivity to active or inactive excipients of fulvestrant, aromatase inhibitors or castor oil

8. Any severe concomitant condition which makes it undesirable for the patient to participate in the trial or which would jeopardize compliance with the trial protocol. eg., uncontrolled cardiac disease or uncontrolled diabetes mellitus.

3.3.4 Restrictions

1. Patients who are blood donors should not donate blood during the study and for 12 weeks following their last dose of study treatment

2. Patients who have confirmed disease progression must be discontinued from their randomised treatment

3. Concomitant treatments listed in Section 3.7.

3.3.5 Discontinuation of patients from treatment or assessment

3.3.5.1 Criteria for discontinuation

Patients may be discontinued from study treatment and assessments at any time. Specific reasons for discontinuing a patient from this study are:

- Voluntary discontinuation by the patient who are at any time free to discontinue their participation in the study, without prejudice to further treatment
- Safety reasons as judged by the investigator and/or AstraZeneca (Adverse event)
- Severe non-compliance to protocol as judged by the investigator and/or AstraZeneca
- Incorrect enrolment (ie, the patient does not meet the required inclusion/exclusion criteria) of the patient
- Progression of disease
- Lost to follow-up
- Any other reasons not listed above as per investigator discretion. The reason must be adequately documented.

Patients who elect not to receive further study treatment will continue to have objective tumour assessments until disease progression, unless they withdraw their consent from collection of data beyond the point of withdrawal from study treatment.

Patients who withdraw their consent for study participation will no longer receive any protocol mandated assessments. Patient data will not be collected beyond the date of consent withdrawal.

3.3.5.2 Procedures for discontinuation

Patients who discontinue should always be asked about the reason(s) for their discontinuation and about the presence of any adverse events. If possible, they should be seen and assessed by an investigator. Adverse events should be reported for up to 8 weeks after the last injection

for patients randomised to fulvestrant or 30 days after the last tablet for patients randomised to anastrozole. The patient should return any investigational products.

The reason for withdrawal and the date of withdrawal from the study must be documented on the CRF provided. If the patient withdraws from the study every effort should be made to measure the tumour at the time of withdrawal. If a patient discontinues randomised treatment prior to disease progression, and does not withdraw consent, they should continue to be followed up for progression. (This is not required after the final DCO for the final analysis.)

3.4 Treatments

3.4.1 Identity of investigational product and comparators

AstraZeneca will supply the following study drugs:

Fulvestrant (FASLODEX™) as a 5% w/v solution in clear neutral glass pre-filled syringes (PFS). Each syringe will contain 250 mg of fulvestrant in 5 ml (Formulation Number F6521). The constituents of the solution are as follows: fulvestrant, ethanol 96%, benzyl alcohol, benzyl benzoate and castor oil.

Anastrozole (ARIMIDEX™) 1 mg white film coated tablets (Formulation number F11292). The constituents of each tablet are as follows: anastrozole, lactose monohydrate, macrogol, magnesium stearate, hypromellose, povidone, sodium starch glycollate and titanium dioxide.

Each fulvestrant pre-filled syringe will be packed into a light excluding carton.

Anastrozole 1 mg tablets will be packed into tamper evident, high density polyethylene (HDPE) bottles with an induction seal child resistant cap. Each bottle will contain 35 tablets (sufficient for 28 days plus 7 days overage).

The pre-filled syringes and the tablets will be packaged separately due to different storage conditions and will not be centre or patient specific.

3.4.2 Doses and treatment regimens

Patients will be randomised to receive;

1. Fulvestrant 500 mg im on days 0, 14 (± 3), 28 (± 3) and every 28 (± 3 days) thereafter. Time windows extended to ± 7 days after 24 weeks.

2. Anastrozole 1 mg po once daily.

In calculating days of treatment administration, the first day of treatment is considered as day 0. Therefore, if injections are given on a Monday, the Monday four weeks later will be considered day 28.

Fulvestrant will be provided as 250 mg in 5 ml as a pre-filled syringe. Each dose of fulvestrant will be administered as 500 mg, that is, two 5 ml intramuscular injections, one in

each buttock. Injections will be given as 500 mg on days 0, 14 (± 3), 28 (± 3) and every 28 (± 3 days) thereafter. Time windows extended to ± 7 days after 24 weeks.

Each injection will be administered intramuscularly into the upper outer quadrant of the buttock using aseptic parenteral technique and must be administered slowly over approximately 1-2 minutes. Following administration, the injection sites should be assessed by the investigator for any local reaction. The patient should be instructed to report complications to the investigator. Appropriate measures such as the application of heat or cold should be instituted according to basic nursing intervention and institutional policy and pressure should be applied where appropriate. Any severe local site reaction should be treated with appropriate medical intervention.

Anastrozole 1 mg will be administered as one tablet daily. Each bottle will contain 35 tablets, allowing for 28 days treatment, plus an additional 7 days if a patients' visit is delayed.

Study treatments must be administered within 7 days of randomisation and will continue until progression, or until considered by the investigator not in the best interest of the patient, whichever occurs first. Reasons for treatment discontinuation will be collected.

3.4.3 Labelling

Labelling of the investigational product will be performed in accordance with GMP, Good Manufacturing Practice. The labels used will be produced in the local language, and in accordance with local regulations for each participating country.

Each fulvestrant pre-filled syringe will be labelled with a single panel label and packed into an individual carton labelled with a two-panel label. The left portion of the label will remain on the individual carton. Prior to administration of the 2 doses at each dosing period, the right portion of each tear-off label will be removed from the 2 cartons and affixed to the appropriate CRF pages as part of the patient's permanent record.

The bottles containing the anastrozole 1 mg tablets will be labelled with a two-panel label. The left portion of the label will remain on the bottle. Prior to the dispensing of each bottle of tablets, the right portion of the tear-off label will be removed from the bottle and affixed to the appropriate CRF pages as part of the patient's permanent record.

3.4.4 Storage

All investigational products must be kept in a secure place under appropriate storage conditions.

The storage conditions for the drugs are shown on the Clinical Trial Label affixed to each level of packaging.

3.4.5 Accountability

For US centres only, the investigator or the sub-investigators named on the Food and Drug Administration (FDA) Form-1572 will prescribe investigational materials. For centres in

other countries, the investigator or his/her representative will prescribe investigational materials. Under no circumstances will the investigator allow the investigational drug to be used other than as directed by the protocol without prior AstraZeneca written approval.

The investigator must maintain accurate records accounting for the receipt of the investigational materials (AstraZeneca provides a copy of the Investigational Product Shipping Order/Certificate of Delivery for this purpose) and for the disposition of the material. This record keeping consists of a dispensing record including the identification of the patient to whom the drug is dispensed, the quantity and the date of dispensing, and any unused drug returned to the investigator. Patients must return all unused drugs to the investigator. This record is an addition to any drug accountability information.

All patients must return the bottle of tablets monthly to the investigator who will retain these until AstraZeneca designated personnel perform drug accountability and compliance checks. Compliance in taking the tablets will be determined by a recorded tablet count or tablets returned at each clinic visit.

Used pre-filled syringes and returned tablet bottles should be destroyed at the study investigator sites by high temperature incineration or by the standard institutional procedure. Complete, unused study drug will be destroyed by the same method after authorisation by the AstraZeneca designee. The investigator or his representative must sign off on all locally destroyed drugs using the Drug Destruction Form provided. If drug destruction on site is not feasible, the AstraZeneca representative will provide instructions on return of drug. It is essential that the investigator accounts for all drugs, but the task of maintaining accurate records may be delegated to a pharmacist.

3.5 Method of assigning patients to treatment groups

The randomisation scheme will be created by AstraZeneca and will provide the allocation of patient numbers to the treatment regimen in balanced blocks. Complete blocks of randomisation numbers will be assigned to each centre, that is, randomisation will be stratified by centre.

As patients are screened for the study after signing and dating the written Informed Consent they must be allocated a 7-digit enrolment code (E-code) with the prefix ‘E’.

The first four digits in the enrolment code will indicate the centre, and digits 5-7 the enrolment order for the centre (eg., the first patient screened in centre number 0125 would be assigned the E-code E0125001, the second patient screened would be E0125002 and so on).

This number is the patient’s unique identifier and is used to identify the patient on the CRFs. Enrolment numbers should be given in consecutive order. All screened patients are assigned an E-code irrespective of whether or not they are subsequently randomised to receive study treatment.

Patient eligibility will be established before treatment randomisation. Patients will be randomised strictly sequentially, as patients are eligible for randomisation. If a patient discontinues from the study, the patient number will not be reused, and the patient will not be allowed to re-enter the study.

The actual treatment given to individual patients will be determined by the randomisation scheme, and the actual treatments will be prepared and packed by Investigational Product Section, AstraZeneca. For this study randomization cards will be used to allocate treatment to patients. Randomization cards must be allocated to each patient sequentially, and must not be opened until the patient has been deemed eligible for the study. If a randomisation patient code is assigned incorrectly, no attempt should be made to remedy the error once study material has been dispensed. The patient will continue with the allocated randomised patient code and study material. The Astrazeneca representative should be notified as soon as the error is discovered. Randomisation of subsequent patients will continue using the first unallocated randomised patient code in the original sequence.

3.6 Blinding and procedures for unblinding the study (Not applicable)

3.7 Pre-study, concomitant and post-study treatment(s)

All prior treatments for cancer and all drugs given to, or taken by, the patient at entry and during the study must be clearly documented on the appropriate CRF page.

The following restrictions apply:

1. Concomitant anticancer treatments are not permitted during the study. Such treatments are prohibited even if they were given for another indication (eg, megestrol acetate for appetite stimulation, methotrexate for rheumatological disorders)

2. Radiotherapy may be given concomitantly for control of bone pain if therapy was started prior to randomisation. Patients requiring radiation for breast cancer or surgery for a breast cancer site after randomisation will be considered to have progressed, unless the investigator specified otherwise. If the investigator rules out progression, then irradiated or excised lesions will be considered non assessable for response and will be monitored only for disease progression.

3. Bisphosphonate therapy at the time of randomisation *for the management of bone metastases* is recommended as standard of care. If bisphosphonate therapy is initiated after randomisation the reason for its use must be clearly documented. Chronic concomitant bisphosphonate therapy for hypercalcemia, and bisphosphonate treatment for the prevention of bone metastases are not permitted during the study. Bisphosphonate therapy for the treatment of osteoporosis is permitted during the study

4. Sex hormone containing drugs such as hormone-replacement therapy (HRT), progestational agents (megestrol acetate), DHEA, other androgens (eg,

oxandrolone) and SERMs (eg raloxifene (Evista®)) are not permitted during the study. In cases where patients suffer severe menopausal symptoms, management with non-hormonal agents, eg, clonidine or venlafaxine, is recommended. In cases of atrophic vaginitis the use of non-hormonal vaginal moisturizing or lubricating gels or creams is recommended. Use of oestrogen-containing vaginal creams or other topical preparations is not allowed on the study, but use of controlled-release vaginal rings (eg, Estring®) may be considered at the investigator's discretion in severe cases or where all the other treatment possibilities have been exhausted and bearing in mind current advice on their use in combination with aromatase inhibitors.

In addition, other drugs than those mentioned above which may affect sex hormone status or disease response, such as systemic ketoconazole, systemic corticosteriods and adrenocortical suppressants are not allowed to begin after randomisation in to the study. However, the patient can continue to receive such drugs if they were taken before randomisation and the investigator is satisfied that the patient's hormonal status is stable. Hormone antagonists and related agents (eg. soy isoflavones) are not allowed.

Topical applications, inhaled sprays, eye drops, local injections and mouth-washes (if not swallowed) containing corticosteroids or ketoconazole are permitted during the study.

5. Patients receiving long-term anti-coagulant therapy with warfarin are ineligible for the study unless they are receiving low dose warfarin and have an INR ≤1.6. The INR should be checked to ensure that it is ≤1.6 prior to each injection. If the INR is >1.6, the injections may be withheld until the INR has returned to ≤1.6. It is advised to apply direct pressure to the injection site in these patients.

6. Patients who need to begin anti-coagulant therapy while receiving study treatment may be treated, at the discretion of the investigator, with low molecular weight heparin (LMWH). The LMWH should be temporarily discontinued 12-24 hours prior to each injection and then resumed 12-24 hours later (depending on the particular LMWH used). There is an increased risk of haemorrhage in these patients and the investigator should decide whether that risk is outweighed by the possible benefits of continued treatment. It is advised to apply direct pressure to the injection site in these patients.

   If, in the opinion of the investigator, warfarin is required instead of LMWH, it should be recognized that the risk of intramuscular haemorrhage may be increased. In this situation, the dose of warfarin should be chosen according to the condition being treated and the INR should be monitored. The INR should be checked prior to each injection and the injection may be withheld if the INR is >1.6. It is advised to apply direct pressure to the injection site in these patients.

Anticoagulant therapy should be driven by the indication for which it is being administered. Should temporary cessation of treatment pose a risk to the patient the investigator should decide whether that risk is outweighed by the possible benefits of continued treatment.

7. Patients receiving antiplatelet therapy (ASA, ticlopidine, clopidogrel etc.) may be at increased risk of bleeding from intramuscular injection. The investigator should decide whether that risk is outweighed by the possible benefits of continued treatment. It is advised to apply direct pressure to the injection site in these patients.

Other medication, which is considered necessary for the patient's safety and well-being, may be given at the discretion of the investigator(s). The administration of all medication (including investigational products) must be recorded in the appropriate sections of the case report form (CRF).

3.8 Treatment compliance

Compliance in taking the tablets will be determined by a recorded tablet count at each clinic visit. Returned tablets will be counted by the CRA. The investigator or pharmacy must retain records of the administered pre-filled syringes. The CRA will check these records to confirm the compliance with the protocol administration schedule.

4. MEASUREMENTS OF STUDY VARIABLES AND DEFINITIONS OF OUTCOME VARIABLES

4.1 Primary variable

The primary variable for this study is clinical benefit (CB = CR + PR + SD ≥ 24 weeks defined by RECIST criteria).

4.2 Screening and demographic measurements

Please refer to the Study Plan (Table 1) for the list of procedures and assessments to be performed at screening and their relative timings prior to randomisation.

Before entering the study, patients will be assessed to ensure they meet the eligibility criteria (see Section 3.3.2 and Section 3.3.3). Patients not meeting these criteria should not be entered into the study.

Written informed consent must be obtained prior to any study specific assessments. Procedures that are part of standard of care may occur prior to informed consent is obtained (see Section 8.3).

Each patient will undergo screening procedures within 3 weeks prior to randomisation except for baseline tumour assessments (see below).

The data listed below will be collected on the relevant CRFs:

- Date of birth, sex, and race
- Significant medical and surgical history
- Concurrent medical conditions
- Breast cancer history
- Oestrogen and progesterone receptor status

  Whenever possible, the status of both receptors should be recorded. If the status of one of the receptors is not available historically, if possible a retest should be done for the missing receptor data for the purposes of this study. However, this should not delay the inclusion of a patient if a positive status is available for either ER or PgR; the missing receptor test can be performed later and the result added to the database.

- HER-2 status (where available)
- Previous breast cancer therapy
- Concurrent medications (see Section 3.7). All concomitant medication/treatment will be recorded at entry. All changes will be recorded until the patient stops randomised study treatment.
- Blood pressure, heart rate, weight and height
- ECG
- WHO performance status
- Laboratory Haematology and Biochemistry
- Tumour burden and tumour assessments (as per RECIST criteria (see Appendix C)).
  - Tumour assessment data must be available for confirmation of disease before randomisation. All patients must have a chest X-ray or a computerized tomography (CT)/MRI scan of the chest within 4 weeks before treatment and a screening skeletal survey or isotopic bone scan within 8 weeks before treatment. Patients with an abnormal isotopic bone scan or skeletal survey must have further baseline confirmation with assessment by X-ray (or by CT scan, or magnetic resonance imaging [MRI] as appropriate) for clinical evaluation and tumour assessment within 4 weeks prior to treatment. (A skeletal survey done within 4 weeks of treatment does not require further

confirmation by X-ray). All patients must also have an abdominal CT/MRI scan within 4 weeks before treatment.

- Up to 10 target lesions (no more than 5 lesions per organ) can be selected at screening; these target lesions, which must be measurable (as defined in Appendix C), will be monitored by the Investigator throughout the study, and tumour measurements will be collected.

- Previously irradiated lesions will not be considered measurable unless there is definite progression at such lesions at the screen tumour assessments.

- All other (non-target) lesions will also be monitored throughout the study, and an overall assessment of non-target lesions will be made and recorded as “present”, “present with progression” or “absent”.

Screening data will be used as baseline measurements, except for haematology and biochemistry, which must be repeated before study treatment if study treatment does not commence within 7 days after the screening sample was taken. The most recent assessment before first dosing should be entered onto the Visit 1 CRF pages.

At analysis, eligibility (and hence possible protocol violations) will be based on the Visit 1 data.

Day 0/Visit 1 is the day on which the patient first receives her randomised study treatment (ie, the first day to receive study treatment, not the day of randomisation).

The patient should be treated within 7 days following randomisation.

Subsequent visits should occur within ± 3 days of the protocolled visit times up to 24 weeks. Time windows extended to ± 7 days after 24 weeks. Tumour assessments can occur ± 2 weeks of the specified time point.

4.3 Patient-Reported Outcomes (PROs) (Not applicable)

4.4 Health Economic measurements and variables (Not applicable)

4.5 Pharmacokinetic measurements and variables (Not applicable)

4.6 Efficacy and pharmacodynamic measurement and variables

Following initial randomised trial treatment on day 0, subsequent visits (including day 14) and assessments should occur ± 3 days of the protocolled visit times up to 24 weeks. Time windows extended to ± 7 days after 24 weeks. Tumour assessments can occur ± 2 weeks of the specified time point.

At least one set of films/electronic images must be made at each tumour assessment and stored with the hospital records. Copies of the films/electronic images will be sent at regular intervals to the REC (Response Evaluation Committee) for independent review. An independent panel

(REC) consisting of radiologists/oncologists will perform a review of patients with a complete response (CR), partial response (PR) or stable disease (SD) ≥ 24 weeks. An additional review of patients with progressive disease (PD) may be undertaken to explore any disparity between known progression rates for hormonal therapy in the first line setting and those emerging in this trial. The reviewers will be blinded to treatment but not to the sequence of clinical visits. The REC's decision will be used to corroborate the analysis from the investigators assessment. The investigators assessment will be used for the primary analysis.

Patients will be considered lost to follow-up if they miss their visit and have no information available for more than 24 weeks.

Efficacy for all patients will be assessed by objective tumour assessment every 12 weeks of the initial randomised period using the appropriate method as recommended in Appendix C.

In both treatment periods assessments should continue until progression of disease occurs and all tumour assessments should be repeated at cessation of randomised trial treatment.

All patients will be assessed until evidence of one of the following;

1. Progression as defined in this protocol regardless of any changes in breast cancer therapy after entering the study

2. Death without evidence of disease progression.

For patients with measurable disease the RECIST criteria will be used to determine the CB, the objective tumour response (CR or PR) and TTP as well as the best overall objective tumour response; details are given in Appendix C. The revised (May 1999) WHO definitions (RECIST) for measurable, non-measurable, target, and non-target lesions, and the objective tumour response criteria (CR, PR, SD or progression of disease) are presented in Appendix C.

For patients with bone-only disease, progression will be defined as described in Section 4.6.3.1. Lesions must be assessed using the same method and technique on each occasion. Lesions will be recorded on the CRF page in the same order as they were recorded at screening. Details of any new lesions will also be collected. Response and progression will be calculated in comparison to the baseline tumour measurements obtained before starting treatment.

Tumour markers must not be used to assign progression or objective response (see RECIST, Appendix C).

It is important to follow the assessment schedule as closely as possible because CB is the primary endpoint and biases in analysis can occur if 1 treatment group is examined more often or sooner than the other. If an unscheduled radiological and clinical tumour assessment is performed, and the patient has not progressed, the next scheduled tumour assessment should still be performed at the planned time (as detailed in the study plan). This is in order to

minimise any unintentional bias caused by some patients being monitored at a different frequency than other patients.

Patients who are withdrawn from study treatment for reasons other than disease progression will continue to have objective tumour assessments every 12 weeks until progression is documented. Adherence to the study plans should be observed whenever possible.

4.6.1 Clinical benefit rate (CBR)

4.6.1.1 Methods of assessment

Clinical benefit (CB) will be obtained for those patients who have a best response (as defined by RECIST) of either, CR, PR or SD ≥ 24 weeks as defined by the modified RECIST Criteria (see Section 4.6.3.1)

4.6.1.2 Derivation or calculation of outcome variable

CB rate is defined as the proportion of all randomised patients who have a best objective response of CR, PR or SD ≥ 24 weeks.

4.6.2 Objective response rate (ORR)

4.6.2.1 Methods of assessment

Only patients with measurable disease will be assessed for objective response.

For patients with measurable disease at baseline, the RECIST criteria (see Appendix C) will be used to perform the objective tumour assessments and to categorise best overall objective tumour response for target and non-target lesions. Response will be classified as CR, PR, SD, or progressive disease (PD).

A best response of CR means that the CR criteria are confirmed on one visit and that no evidence of disease progression or death occurs within 4 weeks of disease assessment.

A best response of PR means the PR criteria are confirmed on one visit and that no evidence of disease progression or death occurs within 4 weeks of disease assessment.

For patients with an objective response of CR or PR, confirmations of response by repeat imaging must be performed at 4 weeks (or as soon as possible thereafter) following the date of response.

In the case of SD, follow-up measurements must have met the SD criteria at least once after study entry.

4.6.2.2 Derivation or calculation of outcome variable

A patient's best overall objective tumour response will be determined from the start of treatment until progression and will be used for the summaries of objective tumour response.

Objective tumour response will be determined programmatically based on the RECIST criteria.

ORR is defined as the proportion of all treated patients who have a best response of either CR or PR.

4.6.3 Time to Progression (TTP)

4.6.3.1 Methods of assessment

For patients with measurable disease, the RECIST criteria will be used to determine a patient's TTP (see Appendix C).

According to RECIST a patient is determined to have progressed if they have progression of target lesions, clear progression of existing non-target lesions, or the appearance of one or more new lesions. Progression of target lesions is defined as at least a 20% increase in the sum of the longest diameter (LD) of target lesions taking as references the smallest sum of LD recorded.

In the absence of measurable disease at baseline (as per the RECIST criteria), the following will be considered progression among patients with lytic or mixed (lytic + sclerotic) bone lesions:

- The appearance of one or more new lytic lesions in bone
- The appearance of one or more new lesions outside of bone
- Unequivocal progression of existing bone lesions

For patients with both measurable disease (as per the RECIST criteria), and documented lytic or mixed bone lesions at baseline, the definition of progression will be based on the RECIST criteria in addition to the criteria defined above for patients with lytic or mixed bone lesions without measurable disease.

Note: Pathologic fracture, new compression fracture, or complications of bone metastases will not be considered as evidence of disease progression, unless one of the above-mentioned criteria is fulfilled.

The date of progression is the date of the investigation/procedure (imaging, biopsy, etc) that led to the diagnosis of progression. If more than one investigation/procedure is performed, and assuming that more than one confirms progression, the date of progression is the date when the first investigation/procedure was performed. The date of the progression in the case of a biopsy refers to the date of the biopsy itself and not the date of the pathology report. In the case of more than one procedure, where the first one had unclear results that have been confirmed later, that date of progression is the date of the investigation/procedure with clear, definitive results. Progression should not be backdated to the earlier procedure. In the few cases where progression is based solely on clinical evidence, an explanation should be

available in the medical notes at the hospital or clinic, and the date of progression, to the best knowledge of the physician, should be documented. If a patient dies prior to reporting progression, the date of progression will be considered as the date of death.

4.6.3.2 Derivation or calculation of outcome variable

TTP is defined as the time from randomisation to the time of the earliest evidence of objective disease progression or death from any cause prior to documented progression. Death will be regarded as a progression event in those patients who die without evidence of disease progression.

Patients who have not progressed or died at the time of the primary data cut-off date or who have been lost to follow-up will be right-censored at the date of their last RECIST disease assessment.

After the primary DCO for this trial, a further analysis of TTP will be performed. At this point, TTP will be calculated using date of progression (via RECIST) for patients who progressed prior to the primary analysis of this trial and date of progression (investigator's opinion) for patients who did not progress prior to the primary analysis.

In addition, TTF, calculated as time from randomisation until the subject stopped receiving randomised treatment, will also be analysed.

An analysis of OS survival will be performed for all randomised patients when approximately 133 (65%) of patients have died. OS is defined as the number of days from randomisation to death due to any cause. Patients who are not known to have died (including those who have been lost to follow-up with no information on survival) will be right-censored at their last contact date. For patients who are lost to follow-up but for whom a date of death is known, then the date of death will be used in the calculation and analysis of OS.

4.6.4 Duration of response (DoR)

4.6.4.1 Methods of assessment

DoR will be calculated for those patients with measurable disease who have a best response of CR or PR based on the RECIST criteria.

4.6.4.2 Derivation or calculation of outcome variable

DoR will be defined in 2 ways: (1) from date of first documentation of response until the date of disease progression or death from any cause and (2) from the date of randomisation until the date of disease progression or death from any cause. Any patient who has not progressed or died by the date of data cut-off, or who have been lost to follow-up, will be right-censored in the analysis at the date of the their last **RECIST** disease assessment.

4.6.5 Duration of Clinical Benefit (DoCB)

4.6.5.1 Methods of assessment

Duration of clinical benefit will be calculated for all patients who have a best response of CR, PR or SD ≥ 24 weeks.

4.6.5.2 Derivation or calculation of outcome variable

Duration of clinical benefit will be defined from the date of randomisation until the date of disease progression or death from any cause. Any patient who has not progressed or died by the date of data cut-off, or who have been lost to follow-up, will be right-censored in the analysis at the date of the their last **RECIST** disease assessment.

4.6.6 Evaluation of serum-based disease tumour markers

4.6.6.1 Methods of assessment

It is proposed that a 10 ml blood sample be taken pre-treatment, at 4, 12 weeks , 24 weeks and at treatment discontinuation. These samples will be collected, prepared by separating the blood serum, and dividing the serum into 1 ml aliquots, and shipped to the central laboratory storage facility. Complete sample collection and handling instructions will be included in the Laboratory Handbook for Investigators. The serum samples will be archived and evaluated for a panel of existing tumour markers (to possibly include, but not be limited to CA15-3, CA27.29, ECD-HER-2/neu and CEA), as well as markers yet to be determined in order to assess their prognostic and/or predictive value of treatment response and clinical outcome.

4.6.6.2 Derivation or calculation of outcome variable

The evaluation of tumour markers in this population is evolving and a collection of archived material would provide a significant resource for the retrospective assessment of new, and hopefully improved, serum tumour marker assays leading to the generation of further informative data with regard to the drugs being investigated in this clinical trial.

The collection and storage of sequential serum samples from recruited individuals would allow the future testing of novel serum tumour marker assays with comparison to the current 'gold standard' assays. Therefore for the purpose of this trial, no patient management decisions will be made based on these data. The serum tumour marker samples will be stored for no longer than the duration of the study.

4.7 Safety measurements and variables

The methods for collecting safety data are described below.

4.7.1 Adverse events

4.7.1.1 Definitions

The definitions of adverse events (AEs), serious adverse events (SAEs) and other significant adverse events (OAEs) are given below. It is of the utmost importance that all staff involved

in the study are familiar with the content of this section. The principal investigator is responsible for ensuring this.

Adverse event

An adverse event is the development of an undesirable medical condition or the deterioration of a pre-existing medical condition following or during exposure to a pharmaceutical product, whether or not considered causally related to the product. An undesirable medical condition can be symptoms (eg, nausea, chest pain), signs (eg, tachycardia, enlarged liver) or the abnormal results of an investigation (eg, laboratory findings, electrocardiogram). In clinical studies, an AE can include an undesirable medical condition occurring at any time, including run-in or washout periods, even if no study treatment has been administered.

Any events that are unequivocally because of progression of the disease under study must not be reported as an AE. Adverse events of bone pain that are not considered to be due to disease progression should be reported as an adverse event.

Serious adverse event

A serious adverse event is an AE occurring during any study phase (ie, run-in, treatment, washout, follow-up), and at any dose of the investigational product or comparator that fulfils one or more of the following criteria:

- results in death
- is immediately life-threatening
- requires in-patient hospitalisation or prolongation of existing hospitalisation
- results in persistent or significant disability or incapacity
- is a congenital abnormality or birth defect
- is an important medical event that may jeopardise the patient or may require medical intervention to prevent one of the outcomes listed above.

The causality of SAEs (ie, their relationship to study treatment) will be assessed by the investigator(s), who in completing the relevant case report form must answer “yes” or “no” to the question “Do you consider that there is a reasonable possibility that the event may have been caused by any of the following – study medication – other medication?”. For further guidance on the definition of a SAE and a guide to the interpretation of the causality question, see Appendix B to the Clinical Study Protocol. **Any serious events that are unequivocally because of progression of disease must not be reported as an SAE.**

Note that SAEs that could be associated with any study procedure should also be reported. For such events the causal relationship is implied as “yes”.

Other Significant Adverse Events (OAE)

OAEs will be identified by the Drug Safety Physician and if applicable also by the Clinical Study Team Physician during the evaluation of safety data for the Clinical Study Report. Significant adverse events of particular clinical importance, other than SAEs and those AEs leading to discontinuation of the patient from study treatment, will be classified as OAEs. Examples of these are marked haematological and other laboratory abnormalities, and certain events that lead to intervention (other than those already classified as serious), dose reduction or significant additional treatment. For each OAE, a narrative may be written and included in the Clinical Study Report.

4.7.1.2 Recording of adverse events

Any detrimental change in a patient's condition subsequent to their entering the study should be considered an AE.

(i) Method of detecting AE/SAEs

At each visit the method of detecting AEs and SAEs will be by:

(a) information volunteered by the patient or carer

(b) open-ended and non-leading verbal questioning of the patient at every visit such as the following: How are you feeling? Have you had any (other) medical problems since your last visit?

(c) observation by the investigational team, other care providers or relatives

(ii) Time period for collection of AEs/SAEs

Non-serious adverse events and SAEs will be collected from the time consent is given, throughout the treatment period and up to 8 weeks after the last injection for patients randomised to fulvestrant, or 30 days after the last tablet for patients randomised to anastrozole.

During the follow-up phase, only SAEs will be collected (for patients receiving randomised treatment).

(iii) Collection of AE data

All AEs will be recorded on the CRFs provided. A description of the event, including its date of onset and resolution, whether it constitutes a SAE or not, any action taken (eg, changes to study treatment, other treatment given, and follow-up tests) and outcome, should be provided along with the investigator's assessment of causality (the relationship to the study treatment). AEs will also be graded according to the NCI CTC version 3.

(iv) Causality

For an AE to be a suspected drug-related event, there should be at least a reasonable possibility of a causal relationship between the study medicinal product and the AE (see Appendix B for guidelines on interpretation of causality).

(v) Intensity

It is important to distinguish between serious and severe AEs. Severity is a measure of intensity whereas seriousness is defined by the criteria in Section 4.7.1.1. An AE of severe intensity need not necessarily be considered serious. For instance nausea that persists for several hours may be considered severe nausea, but not an SAE. On the other hand, a stroke that results in only a limited degree of disability may be considered a mild stroke but would be an SAE.

The degree of severity will be recorded by means of the National Cancer Institute, Common Toxicity Criteria (CTC) criteria (version 3) for all events with an assigned CTC grade. Where a CTC grade is not available for a particular event, a CTC grade should be assigned to the event by applying the same principles and criteria (1=mild, 2=moderate etc.)

(vi) Disease progression

Where there is deterioration in the condition for which the study treatment is being used, there may be uncertainty as to whether this is lack of efficacy or constitutes an AE. In such cases, unless the AstraZeneca or reporting physician considers that the study treatment contributed to the deterioration, or local regulations state to the contrary, the deterioration should be considered to be disease progression and not an AE. Expected progression should not be reported as an AE, as events of disease progression/lack of efficacy are captured elsewhere in the CRF. However, signs and symptoms of the disease/disorder being studied that are more severe in intensity or more frequent than expected should be reported as AEs.

Any events that are unequivocally due to progression of disease must **not** be reported as an AE.

(vii) Adverse events related to elective surgery or surgery for breast cancer

A hospitalisation for breast cancer, or elective surgery for a medical condition existing prior to the study that has not worsened during the study should not be recorded as an adverse event or as a serious adverse event. However, complications of surgery should be recorded as adverse events or as serious adverse events if they fulfil any of the criteria for seriousness (see Section 4.7.1.1).

(viii) Deaths

All deaths that occur during the study, or within the protocol defined follow-up period after the last injection or tablet of study treatment, must be reported as follows:

Death, unequivocally the result of disease progression should be reported to the study monitor at the next monitoring visit and should be documented in the CRF, but should not be reported as an SAE.

Where death is not due (or not clearly due) to progression of the disease under study, the AE causing the death must be reported to the study monitor (or for US sites to the AZ Study Team) as an SAE within 24 hours. The report should contain a comment regarding the co-involvement of progression of disease, if appropriate, and should assign main and contributory causes of death.

Where the cause of death is not reported, the investigator should where possible pro-actively follow up to establish the cause of death and if possible obtain autopsy results.

(ix) New cancers

The development of a new cancer should be regarded as an AE. New cancers are those that are not the primary reason for the administration of the study treatment and have been identified after the patient's inclusion in this study. They do not include metastases of the original cancer. Symptoms of metastasis or the metastasis itself should not be reported as an AE/SAE as they are considered to be disease progression.

(x) Abnormal laboratory values/vital signs

The reporting of laboratory values/vital signs abnormalities as both laboratory findings and adverse events should be avoided. It is recommended that laboratory value/vital sign abnormalities are not recorded as adverse events unless:

- The abnormal laboratory value/vital sign fulfils any of the criteria for a serious adverse event (SAE), or
- The patient is withdrawn from/discontinues the study as a result of the abnormal laboratory/vital sign, or
- At the investigator's discretion, the laboratory value/vital sign abnormality is deemed to be significant in it's own right and as such considers it necessary to record the abnormality as an adverse event. However, the investigator should be aware that laboratory/vital signs values outside of the normal reference range will be captured elsewhere.

If an abnormal laboratory value /vital sign is associated with clinical signs and symptoms, the sign/symptom should be reported as an AE and the associated laboratory result/vital sign should be considered additional information that must be collected on the relevant CRF module.

(xi) Pregnancy

Only postmenopausal women are eligible to participate in this study (see Section 9.4). However, if for any reason a pregnancy occurs it should be reported to AstraZeneca immediately using specific pregnancy reporting forms.

(xii) Overdose

An overdose is defined as a dose administered to a subject that is in excess of the randomised dose for that patient.

Should an overdose (accidental or deliberate) occur, it must be reported in accordance with the procedures described in Section 9.3. All symptoms associated with the overdose should be reported as AEs.

AEs that occur in association with an overdose should be reported in the same manner as other AEs, following the procedures described in the protocol.

(xiii) Follow up of AEs/SAEs

After the initial AE/SAE report, the investigator is required to follow up proactively each patient and provide further information to AstraZeneca on the patient's condition. During the study all AEs/SAEs should be followed up to resolution, or until the condition stabilises, unless the event is considered by the investigator to be unlikely to resolve due to the patient's underlying disease, or the patient is lost to follow up.

(xiv) Handling unresolved AE/SAEs at completion/withdrawal

Any SAEs that are ongoing when the patient completes the study, or at patient discontinuation from the study, must be followed until resolution or until the patient is lost to follow up, unless, in the investigator's opinion, the condition is unlikely to resolve due to the patient's underlying disease.

AstraZeneca reserves the right to ask for further information on any AE, which may be considered of interest.

(xv) Interaction

Current experience with fulvestrant has not shown potential for drug interaction.

However, for the purpose of this study, if, in the opinion of the investigator, an interaction between fulvestrant and a concomitant drug or between concurrent drugs has occurred, this should be reported as an adverse event. Information should be provided as to which drugs the interaction has occurred between. Any adverse event that occurs as a consequence of the interaction and fulfils the criteria for seriousness must be reported to AstraZeneca immediately, on the same day as per other serious adverse events. Adverse events (serious and non serious) arising as the result of an interaction should be recorded on an adverse event form with an indication that the event is a result of an interaction. For example 'hypotension related to drug-drug interaction'.

Where a drug-drug interaction is reported the investigator should specify which drugs the suspected interaction was between.

4.7.1.3 Reporting of serious adverse events

Investigators and other site personnel must inform the appropriate AstraZeneca representatives of any SAE that occurs in the course of the study within 1 day (ie, immediately but no later than the end of the next business day) of when he or she becomes aware of it.

The Astrazeneca representative will work with the investigator to compile all the necessary information and ensure that the relevant AstraZeneca Clintrace Data Entry Site (CDES) receives a report by day one for all fatal and life-threatening cases and by day five for all other SAEs.

Within the same time frames, the investigator must also report all follow-up information or corrections to data previously submitted on SAEs.

If a non-serious AE becomes serious, this and other relevant follow-up information **must** also be provided to AstraZeneca within 1 day as described above.

All SAEs have to be reported, whether or not considered causally related to the investigational product or to the study procedure(s). All SAEs will be recorded in the pCRF. The investigator is responsible for informing the Ethics Committee and/or the Regulatory Authority of the SAE as per local requirements.

The adverse event dictionary used at the beginning of this study will be MedDRA. Any new versions released will be implemented as appropriate.

4.7.2 Laboratory safety measurements and variables

4.7.2.1 Methods of assessment

During the conduct of this study, will be utilised for the analysis of haematology and biochemistry values in order to assure standardisation of assessments and meaningful comparisons between the two treatment arms. However, the local laboratory in each centre may be used to perform any laboratory assessments required by the investigator as part of the routine medical management of the patient.

For the purpose of assessing eligibility, patients may be screened within 3 weeks prior to randomisation by a local laboratory. However, all patients must have a baseline haematology and biochemistry evaluation performed by within 7 days prior to treatment. For participating centres worldwide, will be performing all laboratory safety measurements for this study. For centres in countries where use of is not possible, a local Central Laboratory may be used if available, if not, a centre-specific local laboratory may be used.

may be used to determine eligibility if performed in time to allow review of the results within 7 days before randomisation and treatment. Results from the local

laboratory may be used to determine eligibility should the investigator wish to treat the patient prior to the receipt of the results from the central laboratory. Further samples will be assessed at week 4 and week 12, and at 12-week intervals thereafter, until withdrawal from randomised treatment.

will supply all venepuncture equipment, sample containers and labels. Procedures for sampling, handling and shipping of these samples will be provided in the 'Laboratory Handbook for Investigators' to be provided to each site.

Biochemistry

Fasting is not required for the biochemistry assessments

A 6 ml (maximum) blood sample will be taken into a serum-separator tube and assessed for the following:

Creatinine
Total bilirubin
AP
AST
ALT

Haematology

A 4.5-ml (maximum) blood sample will be taken into an EDTA-coated tube and assessed for the following: red blood cell count, haemoglobin, mean cell volume, platelet count, white blood cell count (total) and lymphocyte and neutrophil counts (in both cases, percentage of white cells and absolute count).

An estimate of the total volume of blood that will be drawn during the study for each patient is shown in Table 4 (See Section 4.8).

4.7.2.2 Derivation or calculation of outcome variables

Section 4.7.1.2(x) provides details of how AEs based on laboratory tests will be recorded and reported.

4.7.3 Vital signs, ECG and physical examination

4.7.3.1 Methods of assessment

Vital signs, physical examinations (including WHO performance status) will be performed at visits 1, 4, 6, 8 and every 12-week visit thereafter, up to and including treatment discontinuation.

ECG is only recorded routinely at screening and in the event of a cardiac adverse event. ECG will be evaluated locally. If a cardiac event should occur, an ECG should be performed and the results recorded on the relevant CRF page. The same method of assessment should be

used throughout. Any clinically significant abnormal findings observed during the study will be recorded as AEs.

WHO performance status will be recorded as follows:

0 = Fully active, able to carry out all usual activities without restrictions and without the aid of analgesia

1 = Restricted in strenuous activity, but ambulatory and able to carry out light work or pursue a sedentary occupation. This group also contains patients who are fully active, as in Grade 0, but only with the aid of analgesics

2 = Ambulatory and capable of all self-care, but unable to work. Up and about more than 50% of waking hours

3 = Capable of only limited self-care, confined to bed or chair more than 50% of waking hours

4 = Completely disabled, unable to carry out any self-care and confined totally to bed or chair.

4.7.3.2 Derivation or calculation of outcome variables

Any new medical condition reported during the study will be recorded as an AE. Only those findings that are in addition to the condition being treated will be recorded as AEs (see Section 4.7.1.2 for recording of AEs). Conditions that are considered by the investigator to be unequivocally disease-related will not be recorded as AEs.

4.8 Volume of blood sampling and handling of biological samples

The total volume of blood drawn from each patient will depend on the length of time the patient receives study medication. Table 4 is a guide to the approximate volume of blood that will be drawn from each patient, based on the assumption that each patient will receive study treatment for 36 weeks.

Table 4 Volume of blood to be drawn from each patient

Assessment		Sample volume (mL)	No. of samples	Total volume (mL)
Safety	Biochemistry	6	6	36
	Haematology	4.5	6	27
	Serum-based disease tumour markers	10	4	40
**Total**				103

4.8.1 Analysis of biological samples

The analyte stability limits defined by the central laboratory will be applied to all analyses performed on behalf of AstraZeneca. The central laboratory will not analyse samples that fall outside these stability limits. Analytical data will not be reported if found to have been derived from a sample that fell outside these stability limits. The standards of procedure followed by the central laboratory may be amended in accordance with its Standard Operating Procedures. The central laboratory will inform AstraZeneca of the stability limits relevant to this study before the first patient gives informed consent to take part in the study.

If the central laboratory chooses to sub-contract the analytical work to another laboratory, the laboratory must assure itself and provide assurance to AstraZeneca that the other laboratory will apply defined stability limits to all analyses performed on behalf of AstraZeneca. Samples falling outside these limits must not be analysed or data reported. The other laboratory will inform AstraZeneca of the stability limits relevant to this study before the first patient gives informed consent to take part in the study.

5. DATA MANAGEMENT

Case report forms will be provided for the recording of data. The forms will be 3 level NCR (no carbon required) paper. Data is to be recorded legibly onto the case record forms in black or blue ballpoint ink. Corrections should be made legibly and initialled and dated by approved personnel; the reasons for significant changes must be provided. Correction fluid or covering labels must not be used. The top original and 1st copy of each completed form will be collected. The top original will be sent to data management personnel in AstraZeneca, the 1st copy will be retained by the monitor. The 2nd copy will be retained at the investigator site.

The method of distribution of data queries will be documented in the study Data Management Plan. The original signed data query will be returned to AstraZeneca. The monitor will retain

one copy and the other retained at the investigator site. On receipt of the data query by data management in AstraZeneca the database will be edited appropriately.

After the primary analysis, all the data entry and data management activities for the follow-up phase of the study were transitioned to the AstraZeneca Data Management Centre (DMC), managed by . The overall survival data will be also be collected on paper CRFs, and sent to the DMC for data entry and validation.

Any electronic data loaded onto the database will be fully checked and validated according to agreed data management guidelines and standards.

6. STATISTICAL METHODS AND DETERMINATION OF SAMPLE SIZE

6.1 Statistical evaluation – general aspects

A comprehensive Statistical Analysis Plan (SAP) will be prepared before database lock **both for the primary analysis and for the analysis of the follow-up phase.**.

6.2 Description of outcome variables in relation to objectives and hypotheses

The primary objective of this study is to compare the clinical benefit rate of patients treated with fulvestrant 500 mg with the clinical benefit rate of patients treated with anastrozole 1 mg.

The secondary objectives of the study are:

- **to compare the objective response rate of patients treated with fulvestrant 500 mg with the objective response rate of patients treated with anastrozole 1 mg**
- **to compare the time to progression of patients treated with fulvestrant 500 mg with the time to progression of patients treated with anastrozole 1 mg**
- **to describe the duration of response of patients treated with fulvestrant 500 mg and the duration of response of patients treated with anastrozole 1 mg**
- **to describe the duration of clinical benefit of patients treated with fulvestrant 500 mg and the duration of clinical benefit of patients treated with anastrozole 1 mg**
- **to assess the safety and tolerability of fulvestrant 500 mg treatment compared with anastrozole 1 mg treatment.**

The exploratory objectives of the study are:

- **to explore the best overall response to the first subsequent systemic breast cancer therapy for patients randomised to fulvestrant 500 mg and patients randomised to anastrozole 1 mg**

- **to evaluate subsequent clinical outcome in patients demonstrating changes in serum tumour markers for patients randomised to fulvestrant 500 mg and patients randomised to anastrozole 1 mg.**

Outcome variables:

- Efficacy

(Primary) - Clinical Benefit (CB = CR + PR + SD ≥ 24 weeks defined by RECIST criteria)

(Secondary) - Objective Response (OR = CR + PR defined by RECIST criteria), Time to Progression (TTP), Duration of Response (DoR), Duration of Clinical Benefit (DoCB)

(Exploratory) – best overall response to subsequent therapy (CR, PR, SD ≥ 24 weeks, SD<24 weeks, PD **or** NE as defined by the Investigator), serum tumour marker levels.

- Safety

Frequency and Severity of Adverse Events as assessed by CTC grade and laboratory assessments

6.3 Description of analysis sets

The primary statistical analyses of the efficacy endpoints will be on an intention-to-treat (ITT) basis and will include all randomised patients. Comparison of treatment groups will be on the basis of randomised treatment, regardless of the treatment actually received. In addition, a per protocol (PP) analysis excluding significant protocol violators and deviators will be carried out for the primary analysis of clinical benefit.

The safety and tolerability data for this study will be summarised according to treatment received. The analysis population will comprise all patients who received at least one dose of fulvestrant or anastrozole.

6.4 Method of statistical analysis

6.4.1 Clinical benefit (CB)

CBR is defined as the proportion of responders (Best objective response of CR or PR) plus those with SD ≥ 24 weeks. A treatment comparison of CBR will be performed using a logistic regression model with treatment factor only. The results will be expressed in terms of the odds ratio together with the corresponding 95% CI and p-value. The estimate of the

difference in CBR rates (fulvestrant – anastrozole) and the corresponding 2-sided 95% confidence interval will also be presented.

6.4.2 Objective response (OR)

OR rate is defined as the proportion of responders (Best objective response of CR or PR).

A point estimate of ORR and the corresponding 2-sided 95% CI will be calculated for each treatment group.

An analysis of OR rate will be performed using a logistic regression (as for CBR). In addition, the best objective response of CR, PR, SD ≥24 weeks, SD <24 weeks, PD or NE will be summarised for each treatment group.

6.4.3 Time to progression (TTP) and Time to treatment failure (TTF)

TTP will be summarised using the Kaplan-Meier method. Kaplan-Meier plots and Kaplan-Meier estimates of median time to progression will be presented for each treatment group. The primary analysis method for TTP will be the log-rank test. The comparison of the treatment groups will be estimated using the hazard ratio of fulvestrant to anastrozole together with corresponding 95% CI and p-value.

TTP and TTF after the DCO for the final analysis will be summarised and analysed for TTP and TTF using the same methods as above. In addition, a secondary analysis method will be performed. The secondary analysis will be the Cox proportional hazards regression model, with treatment factor and the baseline covariates of age, receptor status at diagnosis, visceral involvement, measurable disease and prior chemotherapy.

6.4.4 Duration of response (DoR)

Duration of response will be summarized in 2 ways (see Section 4.6.4.2) using the Kaplan-Meier method. Kaplan-Meier plots and Kaplan-Meier estimates of the median DoR will be presented for the responders in each treatment group.

6.4.5 Duration of clinical benefit (DoCB)

Duration of clinical benefit will be summarized from the date of randomisation until the date of disease progression or death from any cause (see Section 4.6.5.2) using the Kaplan-Meier method. Kaplan-Meier plots and Kaplan-Meier estimates of the median DoCB will be presented for the responders in each treatment group.

6.4.6 Safety and tolerability

Safety and tolerability variables will be summarised by treatment actually received. AE data will be summarised in MedDRA by preferred term and system organ class. This will include a summary of patients who experience SAEs, withdraw due to AEs or die due to AEs.

6.4.7 Exploratory analysis

The best objective response (CR, PR, SD ≥24 weeks, SD <24 weeks, PD or NE) to the first subsequent systemic breast cancer therapy after withdrawal from randomised treatment will be summarised by randomised treatment group. The levels of serum tumour markers will be listed and summarised by treatment group. The relationship between serum tumour markers and clinical outcome will be explored.

Full details of the analyses will be provided in the Statistical Analysis Plan which will be finalised before database lock.

6.4.8 Overall Survival

OS will be summarised using the Kaplan-Meier method as described for TTP. Estimates of the median time to death will be presented for each treatment group and a Kaplan-Meier plot will be produced. The primary analysis method for OS will be the log-rank test. The comparison of the treatment groups will be estimated using the hazard ratio of fulvestrant to anastrozole together with the corresponding 95% CI and p-value. In addition, a secondary analysis of OS will be performed. The secondary analysis method will be the Cox proportional hazards regression model with treatment factor and the baseline covariates of age, receptor status at diagnosis, visceral involvement, measurable disease and prior chemotherapy.

6.5 Determination of sample size

The primary end-point is clinical benefit. The clinical benefit rate for anastrozole in HR+ patients is estimated as 60% from previous studies of anastrozole in this patient population (Studies 1033IL/0027 and 1033IL/0030; Bonneterre et al, 2000and Bonneterre et al, 2001; Nabholtz et al, 2000and Nabholtz et al, 2003). One hundred randomised patients per treatment group (200 total) would be required to give 80% power to rule out an absolute deficiency of 20% in clinical benefit rate for fulvestrant 500 mg; ie, 2-sided 95% confidence interval to exclude a 20% deficiency.

6.6 Interim analyses (Not applicable)

6.7 Data and safety monitoring board (Not applicable)

7. STUDY MANAGEMENT

7.1 Monitoring

Before first patient into the study, a representative of AstraZeneca will visit the investigational study site to:

- determine the adequacy of the facilities

- discuss with the investigator(s) (and other personnel involved with the study) their responsibilities with regard to protocol adherence, and the responsibilities of AstraZeneca or its representatives. This will be documented in a Clinical Study Agreement between AstraZeneca and the investigator

During the study, a monitor from AstraZeneca or company representing AstraZeneca will have regular contacts with the study site, including visits to:

- provide information and support to the investigator(s)
- confirm that facilities remain acceptable
- confirm that the investigational team is adhering to the protocol, that data are being accurately recorded in the CRFs, and that investigational product accountability checks are being performed
- perform source data verification (a comparison of the data in the CRFs with the patient's medical records at the hospital or practice, and other records relevant to the study). This will require direct access to all original records for each patient (eg, clinic charts).

The monitor or another AstraZeneca representative will be available between visits if the investigator(s) or other staff at the centre need information and advice.

7.2 Audits and inspections

Authorised representatives of AstraZeneca, a regulatory authority, an Independent Ethics Committee (IEC) or an Institutional Review Board (IRB) may visit the centre to perform audits or inspections, including source data verification. The purpose of an AstraZeneca audit or inspection is to systematically and independently examine all study-related activities and documents to determine whether these activities were conducted, and data were recorded, analysed, and accurately reported according to the protocol, Good Clinical Practice (GCP), guidelines of the International Conference on Harmonisation (ICH), and any applicable regulatory requirements. The investigator should contact AstraZeneca immediately if contacted by a regulatory agency about an inspection at his or her centre.

7.3 Training of staff

The principal investigator will maintain a record of all individuals involved in the study (medical, nursing and other staff). He or she will ensure that appropriate training relevant to the study is given to all of these staff, and that any new information of relevance to the performance of this study is forwarded to the staff involved.

7.4 Changes to the protocol

Study procedures will not be changed without the mutual agreement of the International Co-ordinating Investigators and AstraZeneca.

If it is necessary for the study protocol to be amended, the amendment or a new version of the study protocol (Amended Protocol) must be notified to or approved by each IRB or IEC, and if applicable, also the local regulatory authority, before implementation. Local requirements must be followed.

If a protocol amendment requires a change to a particular centre's Informed Consent Form, then AstraZeneca and the centre's IRB or IEC must be notified. Approval of the revised Informed Consent Form by AstraZeneca and by the IRB or IEC is required before the revised form is used.

AstraZeneca will distribute amendments and new versions of the protocol to each principal investigator(s), who in turn is responsible for the distribution of these documents to his or her IRB or IEC, and to the staff at his or her centre. The distribution of these documents to the regulatory authority will be handled according to local practice.

7.5 Study agreements

The principal investigator at each centre must comply with all the terms, conditions, and obligations of the Clinical Study Agreement for this study. In the event of any inconsistency between this Clinical Study Protocol and the Clinical Study Agreement, the Clinical Study Protocol shall prevail.

7.6 Study timetable and end of study

Before a patient's enrolment in the study and any study-related procedures are undertaken the following should be fulfilled:

- signed Clinical Study Protocol and other agreements between AstraZeneca and the Principal Investigator/Study Site.
- approval of the study by the IRB/IEC
- approval of the study, if applicable, by the regulatory authority.

The first patient is estimated to be recruited by . Recruitment is expected to be completed by the end of . Investigators will be notified by AstraZeneca when recruitment to the study has been completed.

The data cut-off for the primary analysis will be 6 months after the last patient has been recruited and this is estimated to be at the end of .

After the data cut-off for the primary analysis, all patients, regardless of whether they are still receiving randomised treatment, will enter the follow-up phase and be followed as per standard clinical practice (see Study Plan – Follow-up phase [Table 2]). An analysis of data collected during the follow-up phase will be performed when approximately 75% of patients have discontinued randomised study treatment. The endpoints which will be analysed are TTF and TTP.

Following the DCO for the TTF/TTP analysis, the only data that will be collected is OS data for all randomised patients and SAE data for patients who are still receiving active study treatment. All SAE information will be reported directly to the AZ Patient Safety data entry site and will not be additionally held on the clinical database. The study will be closed when the last patient discontinues their randomised treatment or approximately 133 (65%) of patients have died, whichever is the later.

8. ETHICS

8.1 Ethics review

AstraZeneca will provide IECs and Principal Investigators with safety updates/reports according to local requirements.

The final study protocol, including the final version of the Informed Consent Form, must be approved or given a favourable opinion in writing by an IRB or IEC as appropriate. The investigator must submit written approval to AstraZeneca before he or she can enrol any patient into the study.

The Principal Investigator is responsible for informing the IRB or IEC of any amendment to the protocol in accordance with local requirements. In addition, the IRB or IEC must approve all advertising used to recruit patients for the study. The protocol must be re-approved by the IRB or IEC annually, as local regulations require.

This study will be conducted under an FDA IND at centres in the US only, and at each centre in the US, the Principal Investigator is also responsible for providing the IRB with reports of any serious adverse drug reactions from any other study conducted with the investigational product. AstraZeneca will provide this information to the Principal Investigator via the AstraZeneca designee.

Progress reports and notifications of serious adverse drug reactions will be provided to the IRB or IEC according to local regulations and guidelines.

8.2 Ethical conduct of the study

The study will be performed in accordance with ethical principles that have their origin in the Declaration of Helsinki and are consistent with ICH/Good Clinical Practice, applicable regulatory requirements and the AstraZeneca policy on Bioethics.

8.3 Informed consent

The principal investigator(s) at each centre will ensure that the patient is given full and adequate oral and written information about the nature, purpose, possible risk and benefit of the study. Patients must also be notified that they are free to discontinue from the study at any time. The patient should be given the opportunity to ask questions and allowed time to consider the information provided.

The patient's signed and dated informed consent must be obtained before conducting any procedure specifically for the study.

All consent forms should be signed and dated by the investigator or authorised delegate. If the patient is unable to read or write, the consent forms should be signed and dated by the investigator and an independent witness, to indicate that the patient apparently understood the information and consented freely. Access to patient notes for verification and auditing purposes will be required (see Section 7.2), and permission from each patient must be obtained as part of the consent process.

Where patients return on subsequent occasions for procedures to be repeated, formal consent will not be required on each occasion but their willingness to proceed should be checked.

The principal investigator(s) must store the original, signed Informed Consent Form. A copy of the signed Informed Consent Form must be given to the patient.

The AstraZeneca designee must receive a copy of the Consent Form and patient information sheet (if these are separate documents) used at each centre.

Note: For US centres, AstraZeneca must review and approve the proposed Consent Form before initiation of the study. The proposed form must contain a full explanation of the possible risks, alternative treatment options and availability of treatment in case of injury, in accordance with Federal Regulations as detailed in 21CFR20. It should also indicate by signature that the patient, or where appropriate, legal guardian, permits representatives of AstraZeneca, the CRO, and the FDA access to relevant medical records.

If modifications are made according to local requirements, the new version has to be approved by AstraZeneca.

8.4 Patient data protection

The Master Informed Consent Form will incorporate (or, in some cases, be accompanied by a separate document incorporating) wording that complies with relevant data protection and privacy legislation. Pursuant to this wording, patients will authorise the collection, use and disclosure of their study data by the Investigator and by those persons who need that information for the purposes of the study.

The Master Informed Consent Form will explain that study data will be stored in a computer database, maintaining confidentiality in accordance with national data legislation. All data computer processed by AstraZeneca will be identified by randomisation code / study code / initials.

The Master Informed Consent Form will also explain that for data verification purposes, authorised representatives of AstraZeneca, a regulatory authority, an IRB or IEC may require direct access to parts of the hospital or practice records relevant to the study, including patients' medical history.

9. PROCEDURES IN CASE OF EMERGENCY, OVERDOSE OR PREGNANCY

9.1 AstraZeneca emergency contact procedure

In the case of a medical emergency, contact the Study Delivery Team Leader. If the Study Delivery Team Leader is not available, contact the Study Delivery Team Physician at the AstraZeneca Research and Development site shown below.

Role in the study	Name	Address & telephone number

9.2 Procedures in case of medical emergency

The principal investigator(s) is responsible for ensuring that procedures and expertise are available to handle medical emergencies during the study. **A medical emergency usually constitutes an SAE and should be reported as such, see Section 4.7.1.1.**

9.3 Procedures in case of overdose

No specific antidote is available in the event of overdose. Should an episode of overdose occur, routine supportive measures should be taken.

- Use of study medication in doses in excess of that specified in the protocol should not be recorded in the CRFs as an AE of 'Overdose' unless there are associated symptoms or signs.

- An overdose with associated SAEs should be recorded as the SAE diagnosis/symptoms on the relevant AE forms in the CRFs.
- An overdose with associated non-serious AEs should be recorded as the AE diagnosis/symptoms on the relevant AE forms in the CRFs. In addition, the overdose should be reported on the separate AZ "Clinical Study Overdose Report Form."
- An overdose without associated symptoms should not be recorded as an AE in the CRFs. The overdose should be reported on the separate AZ "Clinical Study Overdose Report Form".

9.4 Procedures in case of pregnancy

Only postmenopausal women are eligible to participate in this study. Pregnancy should be ruled out prior to study start in the case of doubt.

Should pregnancy occur it should be reported to AstraZeneca immediately using the specific pregnancy reporting forms (see Section 4.7.1.2 (xi)).

10. REFERENCES

Addo et al 2002
Addo S, Yates RA, Laight A. A phase I trial to assess the pharmacology of the new oestrogen receptor antagonist fulvestrant on the endometrium in healthy postmenopausal volunteers. Br J Cancer 2002; 87: 1354-59.

Beatson GT 1896
Beatson GT. On the treatment of inoperable cases of carcinoma of the mamma: Suggestions for a new method of treatment with illustrative cases. Lancet 1896;2:104-7.

Bonneterre et al, 2001
Bonneterre J, Buzdar A, Nabholtz JM, Robertson JF, Thurlimann B, von Euler M, et al. Anastrozole is superior to tamoxifen as first-line therapy in hormone receptor positive advanced breast carcinoma. Cancer 2001;92:2247-58.

Bonneterre et al, 2000
Bonneterre J, Thürlimann B, Robertson JFR, Krzakowski M, Mauriac L, Koralewski P, Vergote I, Webster A, Steinberg M, von Euler M: Anastrozole versus tamoxifen as first-line therapy for advanced breast cancer in 668 postmenopausal women - results of the TARGET (Tamoxifen or Arimidex Randomised Group Efficacy and Tolerability) study. J Clin Oncol 2000;18:3748-57.

Buzdar AU 1998
Buzdar AU. Anastrozole: a new addition to the armamentarium against advanced breast cancer. Am J Clin Oncol 1998;21:161-166.

Buzdar et al, 2001
Buzdar A, Douma J, Davidson N, Elledge R, Morgan M, Smith R, et al. Phase III, multicenter, double-blind, randomized study of letrozole, an aromatase inhibitor, for advanced breast cancer versus megestrol acetate. J Clin Oncol 2001;19:3357-66.

DeFriend et al, 1994
DeFriend D, Howell H, Nicholson R, et al. Investigation of a new pure antioestrogen (ICI 182780) in women with primary breast cancer. Cancer Res 1994;54:408-414.

Dombernowsky et al, 1998
Dombernowsky P, Smith I, Falkson G, Leonard R, Panasci L, Bellmunt J, et al. Letrozole, a new oral aromatase inhibitor for advanced breast cancer: double-blind randomized trial showing a dose effect and improved efficacy and tolerability compared with megestrol acetate. J Clin Oncol 1998;16:453-61.

Fisher et al, 1998
Fisher B, Costantino JP, Wickerham DL, Redmond CK, Kavanah M, Crothenin WM, et al. Tamoxifen for prevention of breast cancer: report of National Surgical Adjuvant Breast and Bowel Project P-1 study. J Nat'l Cancer Inst 1998;90:1371-88.

Fisher et al, 2001
Fisher B, Anderson S, Tan-Chiu E, et al. Tamoxifen and chemotherapy for axillary node-negative, oestrogen receptor-negative breast cancer findings from the National Surgical Adjuvant Breast and Bowel Project B-23. J Clin Oncol. 2001;19:931-942.

Geisler et al, 2001
Geisler J, Detre S, Bernsten H, et al. Influence of neoadjuvant anastrozole (ARIMIDEX) on intratumoural oestrogen levels and proliferation markers in patients with locally advanced breast cancer. Clin Cancer Res 2001;7:1230-1236.

Howell et al, 2000
Howell A, Osborne CK, Morris C, Wakeling AE. ICI 182,780 (FASLODEX): development of a novel, "pure" anti-estrogen. Cancer 2000;89:817-825.

Howell et al, 2002
Howell A, Robertson JFR, Quaresma Albano J, et al. Fulvestrant, formerly ICI 182,780, is as effective as anastrozole in postmenopausal women with advanced breast cancer progressing after prior endocrine treatment J Clin Oncol, 2002;20:3396-3403.

Kaufmann et al, 2000
Kaufmann M, Bajetta E, Dirix LY, Fein LE, Jones SE, Zilembo N, et al. Exemestane is superior to megestrol acetate after tamoxifen failure in postmenopausal women with advanced breast cancer: results of a phase III randomized double-blind trial – The Exemestane Study Group. J Clin Oncol 2000;18:1399-411.

McPherson et al, 2000
McPherson K, Steel CM, Dixon JM. ABC of breast diseases. Breast cancer – epidemiology, risk factors and genetics. Brit Med J 2000;321:624–628.

Messori et al, 2000
Messori A, Cattle F, Trippoli S, Vaiani M. Survival in patients with metastatic breast cancer: analysis of randomized studies comparing oral non-steroidal aromatase inhibitors versus megestrol. Anticancer Drugs 2000;11:701-706.

Mouridsen et al, 2001
Mouridsen H, Gershanovich M, Sun Y, et al. Superior efficacy of letrozole versus tamoxifen as first-line therapy for postmenopausal women with advanced breast cancer: results of a phase III study of the International Letrozole Breast Cancer Group. J Clin Oncol 2001;19:2596-2506.

Nabholtz et al, 2000
Nabholtz JM, Buzdar A, Pollak M, Harwin W, Burton G, Mangalik A, Steinberg M, Webster A, von Euler M: Anastrozole is superior to tamoxifen as first-line therapy for advanced breast cancer in postmenopausal women: results of a North American multicenter randomised trial. J Clin Oncol 2000;18:3758-7

Nabholtz et al, 2003
Nabholtz JM, Bonneterre J, Buzdar A, Robertson JFR, Thürlimann B for the Arimidex Writing Committee on behalf of the Investigators: Anastrozole (Arimidex) versus tamoxifen as first-line therapy for advanced breast cancer in postmenopausal women: survival analysis and updated safety results. Eur J Cancer 2003;39:1684–9.

Osborne CK et al, 2002
Osborne CK, Pippen J, Jones SE, et al. Double-blind, randomized trial comparing the efficacy and tolerability of fulvestrant versus anastrozole in postmenopausal women with advanced breast cancer progressing on prior endocrine therapy: Results of a North American trial. J Clin Oncol, 2002;20:3386-3395.

Pippen J et al, 2003
Pippen J, Osborne CK, Howell A, Robertson JFR. 'Faslodex'® (fulvestrant) versus Arimidex® (anastrozole) for the treatment of advanced breast cancer: A prospective combined survival analysis of two multicenter trials. Breast Cancer Res Treat 2003;82(Suppl 1):S101,Abs 426.

Robertson JF et al, 2001
Robertson JF, Nicholson RI, Bundred NJ, et al. Comparison of the short-term biological effects of 7□-[9-(4,4,5,5,5-pentafluoropentylsulfinyl)-nonyl]estra-1,3,5, (100-triene-3,17□-diol (Fulvestrant) versus tamoxifen in postmenopausal women with primary breast cancer. Cancer Res 2001;61:6739-6746.

Robertson JF et al, 2002
Robertson JF, Howell A, Abram P et al. Fulvestrant versus tamoxifen for the first-line treatment of advanced breast carcinoma (ABC) in postmenopausal women. Annals Oncol 2002:13(Suppl.5):46

Wakeling AE et al, 1991
Wakeling AE, Dukes M, Bowler J. A potent specific pure anti-oestrogen with clinical potential. Cancer Research 1991;51:3867-3873.

Weibe VJ et al, 1993
Weibe VJ, Osborne CK, Fuqua SAW, DE, Gregoria MW. Tamoxifen resistance in breast cancer. Crit Rev Oncol Hematol 1993:14:173-88.

AstraZeneca

**Clinical Study Protocol: Appendix B**	
Drug Substance	ZD9238 (Fulvestrant)
Study Code	D6995C00006
Appendix Edition Number	1

Appendix B
Additional Safety Information

FURTHER GUIDANCE ON THE DEFINITION OF A SERIOUS ADVERSE EVENT (SAE)

Life threatening

'Life-threatening' means that the subject was at immediate risk of death from the AE as it occurred or it is suspected that use or continued use of the product would result in the subject's death. 'Life-threatening' does not mean that had an AE occurred in a more severe form it might have caused death (eg, hepatitis that resolved without hepatic failure).

Hospitalisation

Out-patient treatment in an emergency room is not in itself a serious AE, although the reasons for it may be (eg, bronchospasm, laryngeal oedema). Hospital admissions and/or surgical operations planned before or during a study are not considered AEs if the illness or disease existed before the subject was enrolled in the study, provided that it did not deteriorate in an unexpected way during the study.

Important medical event or medical intervention

Medical and scientific judgement should be exercised in deciding whether a case is serious in situations where important medical events may not be immediately life-threatening or result in death, hospitalisation, disability or incapacity but may jeopardize the subject or may require medical intervention to prevent one or more outcomes listed in the definition of serious. These should usually be considered as serious.

Simply stopping the suspect drug does not mean that it is an important medical event; medical judgement must be used.

Examples of such events are:

- *Angioedema not severe enough to require intubation but requiring iv hydrocortisone treatment*
- *Hepatotoxicity caused by paracetamol (acetaminophen) overdose requiring treatment with N-acetylcysteine*
- *Intensive treatment in an emergency room or at home for allergic bronchospasm*
- *Blood dyscrasias (eg, neutropenia or anaemia requiring blood transfusion, etc) or convulsions that do not result in hospitalisation*
- *Development of drug dependency or drug abuse.*

A GUIDE TO INTERPRETING THE CAUSALITY QUESTION

The following factors should be considered when deciding if there is a “reasonable possibility” that an AE may have been caused by the drug.

- Time Course. Exposure to suspect drug. Has the subject actually received the suspect drug? Did the AE occur in a reasonable temporal relationship to the administration of the suspect drug?
- Consistency with known drug profile. Was the AE consistent with the previous knowledge of the suspect drug (pharmacology and toxicology) or drugs of the same pharmacological class? OR could the AE be anticipated from its pharmacological properties?
- Dechallenge experience. Did the AE resolve or improve on stopping or reducing the dose of the suspect drug?
- No alternative cause. The AE cannot be reasonably explained by another aetiology such as the underlying disease, other drugs, other host or environmental factors.
- Rechallenge experience. Did the AE reoccur if the suspected drug was reintroduced after having been stopped? AstraZeneca would not normally recommend or support a rechallenge.
- Laboratory tests. A specific laboratory investigation (if performed) has confirmed the relationship?

A “reasonable possibility” could be considered to exist for an AE where one or more of these factors exist.

In contrast, there would not be a “reasonable possibility” of causality if none of the above criteria apply or where there is evidence of exposure and a reasonable time course but any dechallenge (if performed) is negative or ambiguous or there is another more likely cause of the AE.

In difficult cases, other factors could be considered such as:

- Is this a recognised feature of overdose of the drug?
- Is there a known mechanism?

Ambiguous cases should be considered as being a “reasonable possibility” of a causal relationship unless further evidence becomes available to refute this. Causal relationship in cases where the disease under study has deteriorated due to lack of effect should be classified as no reasonable possibility.

AstraZeneca

**Clinical Study Protocol Appendix C**	
Drug Substance	ZD9238 (Fulvestrant)
Study Code	D6995C00006
Appendix Edition Number	1

Appendix C
Objective Tumour Response Criteria (RECIST)

1. INTRODUCTION

The introduction explores the definitions, assumptions, and purposes of tumour response criteria. Below, guidelines that are offered may lead to more uniform reporting of outcomes of clinical trials. Note that although single investigational agents are discussed, the principles are the same for drug combinations, non-investigational agents, or approaches that do not involve drugs.

Tumour response associated with the administration of anticancer agents can be evaluated for at least three important purposes that are conceptually distinct:

- Tumour response as a prospective end point in early clinical trials. In this situation, objective tumour response is employed to determine whether the agent/regimen demonstrates sufficiently encouraging results to warrant further testing. These trials are typically phase II trials of investigational agents/regimens (*see* section 1.2), and it is for use in this precise context that these guidelines have been developed.

- Tumour response as a prospective end point in more definitive clinical trials designed to provide an estimate of benefit for a specific cohort of patients. These trials are often randomised comparative trials or single-arm comparisons of combinations of agents with historical control patients. In this setting, objective tumour response is used as a surrogate end point for other measures of clinical benefit, including time to event (death or disease progression) and symptom control (*see* section 1.3).

- Tumour response as a guide for the clinician and patient or study subject in decisions about continuation of current therapy. This purpose is applicable both to clinical trials and to routine practice (see section 1.1), but use in the context of decisions regarding continuation of therapy is not the primary focus of this document.

However, in day-to-day usage, the distinction among these uses of the term "tumour response" can easily be missed, unless an effort is made to be explicit. When these differences are ignored, inappropriate methodology may be used and incorrect conclusions may result.

1.1 Response outcomes in daily clinical practice of oncology

The evaluation of tumour response in the daily clinical practice of oncology may not be performed according to predefined criteria. It may, rather, be based on a subjective medical judgment that results from clinical and laboratory data that are used to assess the treatment benefit for the patient. The defined criteria developed further in this document are not necessarily applicable or complete in such a context. It might be appropriate to make a distinction between "clinical improvement" and "objective tumour response" in routine patient management outside the context of a clinical trial.

1.2 Response outcomes in uncontrolled trials as a guide to further testing of a new therapy

"Observed response rate" is often employed in single-arm studies as a "screen" for new anticancer agents that warrant further testing. Related outcomes, such as response duration or proportion of patients with complete responses, are sometimes employed in a similar fashion. The utilisation of a response rate in this way is not encumbered by an implied assumption about the therapeutic benefit of such responses, but rather implies some degree of biologic antitumour activity of the investigated agent.

For certain types of agents (ie cytotoxic drugs and hormones), experience has demonstrated that objective antitumour responses observed at a rate higher than would have been expected to occur spontaneously can be useful in selecting anticancer agents for further study. Some agents selected in this way have eventually proven to be clinically useful. Furthermore, criteria for "screening" new agents in this way can be modified by accumulated experience and eventually validated in terms of the efficiency by which agents so screened are shown to be of clinical value by later, more definitive, trials.

In most circumstances, however, a new agent achieving a response rate determined *a priori* to be sufficiently interesting to warrant further testing may not prove to be an effective treatment for the studied disease in subsequent randomised phase III trials. Random variables and selection biases, both known and unknown, can have an overwhelming effect in small, uncontrolled trials. These trials are an efficient and economic step for initial evaluation of the activity of a new agent or combination in a given disease setting. However, many such trials are performed, and the proportion that will provide false-positive results is necessarily substantial. In many circumstances, it would be appropriate to perform a second small confirmatory trial before initiating large resource-intensive phase III trials.

Sometimes, several new therapeutic approaches are studied in a randomised phase II trial. The purpose of randomisation in this setting, as in phase III studies, is to minimise the impact of random imbalances in prognostic variables. However, randomised phase II studies are, by definition, not intended to provide an adequately powered comparison between arms (regimens). Rather, the goal is simply to identify one or more arms for further testing, and the sample size is chosen so to provide reasonable confidence that a truly inferior arm is not likely to be selected. Therefore, reporting the results of such randomised phase II trials should not imply statistical comparisons between treatment arms.

1.3 Response outcomes in clinical trials as a surrogate for palliative effect

1.3.1 Use in non-randomised clinical trials.

The only circumstance in which objective responses in a non-randomised trial can permit a tentative assumption of a palliative effect (ie beyond a purely clinical measure of benefit) is when there is an actual or implied comparison with historical series of similar patients. This assumption is strongest when the prospectively determined statistical analysis plan provides for matching of relevant prognostic variables between case subjects and a defined series of

control subjects. Otherwise, there must be, at the very least, prospectively determined statistical criteria that provide a very strong justification for assumptions about the response rate that would have been expected in the appropriate "control" population (untreated or treated with conventional therapy, as fits the clinical setting). However, even under these circumstances, a high rate of observed objective response does not constitute proof or confirmation of clinical therapeutic benefit. Because of unavoidable and non-quantifiable biases inherent in non-randomised trials, proof of benefit still requires eventual confirmation in a prospectively randomised, controlled trial of adequate size. The appropriate end points of therapeutic benefit for such a trial are survival, progression-free survival, or symptom control (including quality of life).

1.3.2 Use in randomised trials

Even in the context of prospectively randomised phase III comparative trials, "observed response rate" should not be the sole, or major, end point. The trial should be large enough that differences in response rate can be validated by association with more definitive end points reflecting therapeutic benefit, such as survival, progression-free survival, reduction in symptoms, or improvement (or maintenance) of quality of life.

2. MEASURABILITY OF TUMOUR LESIONS AT BASELINE

2.1 Definitions

At baseline, tumour lesions will be categorised as follows: measurable (lesions that can be accurately measured in at least one dimension [longest diameter to be recorded] as ≥ 20 mm with conventional techniques or as ≥ 10 mm with spiral CT scan [see section 2.2]) or nonmeasurable (all other lesions, including small lesions [longest diameter <20 mm with conventional techniques or <10 mm with spiral CT scan] and truly nonmeasurable lesions).

The term "evaluable" in reference to measurability is not recommended and will not be used because it does not provide additional meaning or accuracy.

All measurements should be recorded in metric notation by use of a ruler or calipers. All baseline evaluations should be performed as closely as possible to the beginning of treatment and never more than 4 weeks before the beginning of treatment.

Lesions considered to be truly nonmeasurable include the following: bone lesions, leptomeningeal disease, ascites, pleural/pericardial effusion, inflammatory breast disease, lymphangitis cutis/pulmonis, abdominal masses that are not confirmed and followed by imaging techniques, and cystic lesions.

(*Note:* Tumour lesions that are situated in a previously irradiated area might or might not be considered measurable, and the conditions under which such lesions should be considered must be defined in the protocol when appropriate.)

2.2 Specifications by methods of measurements

The same method of assessment and the same technique should be used to characterise each identified and reported lesion at baseline and during follow-up. Imaging-based evaluation is preferred to evaluation by clinical examination when both methods have been used to assess the antitumour effect of a treatment.

2.2.1 Clinical examination

Clinically detected lesions will only be considered measurable when they are superficial (eg skin nodules and palpable lymph nodes). For the case of skin lesions, documentation by colour photography—including a ruler to estimate the size of the lesion—is recommended.

2.2.2 Chest x-ray

Lesions on chest x-ray are acceptable as measurable lesions when they are clearly defined and surrounded by aerated lung. However, CT is preferable.

2.2.3 CT and MRI

CT and MRI are the best currently available and most reproducible methods for measuring target lesions selected for response assessment. Conventional CT and MRI should be performed with contiguous cuts of 10 mm or less in slice thickness. Spiral CT should be performed by use of a 5-mm contiguous reconstruction algorithm; this specification applies to the tumours of the chest, abdomen, and pelvis, while head and neck tumours and those of the extremities usually require specific protocols.

2.2.4 Ultrasound

When the primary end point of the study is objective response evaluation, ultrasound should not be used to measure tumour lesions that are clinically not easily accessible. It may be used as a possible alternative to clinical measurements for superficial palpable lymph nodes, subcutaneous lesions, and thyroid nodules. Ultrasound might also be useful to confirm the complete disappearance of superficial lesions usually assessed by clinical examination.

2.2.5 Endoscopy and laparoscopy

The utilisation of these techniques for objective tumour evaluation has not yet been fully or widely validated. Their uses in this specific context require sophisticated equipment and a high level of expertise that may be available only in some centres. Therefore, utilisation of such techniques for objective tumour response should be restricted to validation purposes in specialised centres. However, such techniques can be useful in confirming complete histopathologic response when biopsy specimens are obtained.

2.2.6 Tumour markers

Tumour markers alone cannot be used to assess response. However, if markers are initially above the upper normal limit, they must return to normal levels for a patient to be considered in complete clinical response when all tumour lesions have disappeared. Specific additional

criteria for standardised usage of prostate-specific antigen and CA (cancer antigen) 125 response in support of clinical trials are being validated.

2.2.7 Cytology and histology

Cytologic and histologic techniques can be used to differentiate between partial response and complete response in rare cases (eg after treatment to differentiate between residual benign lesions and residual malignant lesions in tumour types such as germ cell tumours). Cytologic confirmation of the neoplastic nature of any effusion that appears or worsens during treatment is required when the measurable tumour has met criteria for response or stable disease. Under such circumstances, the cytologic examination of the fluid collected will permit differentiation between response or stable disease (an effusion may be a side effect of the treatment) and progressive disease (if the neoplastic origin of the fluid is confirmed). New techniques to better establish objective tumour response will be integrated into these criteria when they are fully validated to be used in the context of tumour response evaluation.

3. TUMOUR RESPONSE EVALUATION

3.1 Baseline evaluation

3.1.1 Assessment of overall tumour burden and measurable di sease

To assess objective response, it is necessary to estimate the overall tumour burden at baseline to which subsequent measurements will be compared. Only patients with measurable disease at baseline should be included in protocols where objective tumour response is the primary end point. Measurable disease is defined by the presence of at least one measurable lesion (as defined in section 2.1). If the measurable disease is restricted to a solitary lesion, its neoplastic nature should be confirmed by cytology/histology.

3.1.2 Baseline documentation of "target" and "nontarget" lesions

All measurable lesions up to a maximum of five lesions per organ and 10 lesions in total, representative of all involved organs, should be identified as target lesions and recorded and measured at baseline. Target lesions should be selected on the basis of their size (those with the longest diameter) and their suitability for accurate repeated measurements (either by imaging techniques or clinically). A sum of the longest diameter for all target lesions will be calculated and reported as the baseline sum longest diameter. The baseline sum longest diameter will be used as the reference by which to characterise the objective tumour response.

All other lesions (or sites of disease) should be identified as nontarget lesions and should also be recorded at baseline. Measurements of these lesions are not required, but the presence or absence of each should be noted throughout follow-up.

3.2 Response criteria

3.2.1 Evaluation of target lesions

This section provides the definitions of the criteria used to determine objective tumour response for target lesions. The criteria have been adapted from the original WHO Handbook, taking into account the measurement of the longest diameter only for all target lesions: complete response—the disappearance of all target lesions; partial response—at least a 30% decrease in the sum of the longest diameter of target lesions, taking as reference the baseline sum longest diameter; progressive disease—at least a 20% increase in the sum of the longest diameter of target lesions, taking as reference the smallest sum longest diameter recorded since the treatment started or the appearance of one or more new lesions; stable disease—neither sufficient shrinkage to qualify for partial response nor sufficient increase to qualify for progressive disease, taking as reference the smallest sum longest diameter since the treatment started.

3.2.2 Evaluation of nontarget lesions

This section provides the definitions of the criteria used to determine the objective tumour response for nontarget lesions: complete response—the disappearance of all nontarget lesions and normalisation of tumour marker level; incomplete response/stable disease—the persistence of one or more nontarget lesion(s) and/or the maintenance of tumour marker level above the normal limits; and progressive disease—the appearance of one or more new lesions and/or unequivocal progression of existing nontarget lesions.

(*Note:* Although a clear progression of "nontarget" lesions only is exceptional, in such circumstances, the opinion of the treating physician should prevail and the progression status should be confirmed later by the review panel [or study chair].)

3.2.3 Evaluation of best overall response

The best overall response is the best response recorded from the start of treatment until disease progression/recurrence (taking as reference for progressive disease the smallest measurements recorded since the treatment started). In general, the patient's best response assignment will depend on the achievement of both measurement and confirmation criteria (see section 3.3.1). Table 1 provides overall responses for all possible combinations of tumour responses in target and nontarget lesions with or without the appearance of new lesions.

(*Notes:*

- Patients with a global deterioration of health status requiring discontinuation of treatment without objective evidence of disease progression at that time should be classified as having "symptomatic deterioration." Every effort should be made to document the objective disease progression, even after discontinuation of treatment.
- Conditions that may define early progression, early death, and inevaluability are study specific and should be clearly defined in each protocol (depending on treatment duration and treatment periodicity).

- In some circumstances, it may be difficult to distinguish residual disease from normal tissue. When the evaluation of complete response depends on this determination, it is recommended that the residual lesion be investigated (fine-needle aspiration/biopsy) before confirming the complete response status).

3.2.4 Frequency of tumour re-evaluation

Frequency of tumour re-evaluation while on treatment should be protocol specific and adapted to the type and schedule of treatment. However, in the context of phase II studies where the beneficial effect of therapy is not known, follow-up of every other cycle (ie 6-8 weeks) seems a reasonable norm. Smaller or greater time intervals than these could be justified in specific regimens or circumstances.

After the end of the treatment, the need for repetitive tumour evaluations depends on whether the phase II trial has, as a goal, the response rate or the time to an event (disease progression/death). If time to an event is the main end point of the study, then routine re-evaluation is warranted of those patients who went off the study for reasons other than the expected event at frequencies to be determined by the protocol. Intervals between evaluations twice as long as on study are often used, but no strict rule can be made.

Table 1 Overall responses for all possible combinations of tumour responses in target and nontarget lesions with or without the appearance of new lesions

Target lesions[a]	Nontarget lesions	New lesions	Overall response
CR	CR	No	CR
CR	Incomplete response/StD	No	PR
PR	Non-PD	No	PR
StD	Non-PD	No	StD
PD	Any	Yes or no	PD
Any	PD	Yes or no	PD
Any	Any	Yes	PD

aCR complete response; PR partial response; StD stable disease; and PD progressive disease See text for more details.

3.3 Confirmatory measurement/duration of response

3.3.1 Confirmation

The main goal of confirmation of objective response in clinical trials is to avoid overestimating the response rate observed. This aspect of response evaluation is particularly important in non-randomised trials where response is the primary end point. In this setting, to be assigned a status of partial response or complete response, changes in tumour measurements must be confirmed by repeat assessments that should be performed no less than 4 weeks after the criteria for response are first met. Longer intervals as determined by the study protocol may also be appropriate.

In the case of stable disease, measurements must have met the stable disease criteria at least once after study entry at a minimum interval (in general, not less than 6-8 weeks) that is defined in the study protocol (see section 3.3.3).

(*Note:* Repeat studies to confirm changes in tumour size may not always be feasible or may not be part of the standard practice in protocols where progression-free survival and overall survival are the key end points. In such cases, patients will not have "confirmed response." This distinction should be made clear when reporting the outcome of such studies.)

3.3.2 Duration of overall response

The duration of overall response is measured from the time that measurement criteria are met for complete response or partial response (whichever status is recorded first) until the first date that recurrent or progressive disease is objectively documented (taking as reference for progressive disease the smallest measurements recorded since the treatment started). The duration of overall complete response is measured from the time measurement criteria are first met for complete response until the first date that recurrent disease is objectively documented.

3.3.3 Duration of stable disease

Stable disease is measured from the start of the treatment until the criteria for disease progression is met (taking as reference the smallest measurements recorded since the treatment started). The clinical relevance of the duration of stable disease varies for different tumour types and grades. Therefore, it is highly recommended that the protocol specify the minimal time interval required between two measurements for determination of stable disease. This time interval should take into account the expected clinical benefit that such a status may bring to the population under study.

(*Note:* The duration of response or stable disease as well as the progression-free survival are influenced by the frequency of follow-up after baseline evaluation. It is not in the scope of this guideline to define a standard follow-up frequency that should take into account many parameters, including disease types and stages, treatment periodicity, and standard practice. However, these limitations to the precision of the measured end point should be taken into account if comparisons among trials are to be made.)

3.4 Progression-free survival/time to progression

This document focuses primarily on the use of objective response end points. In some circumstances (eg, brain tumours or investigation of non-cytoreductive anticancer agents), response evaluation may not be the optimal method to assess the potential anticancer activity of new agents/regimens. In such cases, progression-free survival/time to progression can be considered valuable alternatives to provide an initial estimate of biologic effect of new agents that may work by a non-cytotoxic mechanism. It is clear though that, in an uncontrolled trial proposing to utilise progression-free survival/time to progression, it will be necessary to document with care the basis for estimating what magnitude of progression-free survival/time to progression would be expected in the absence of a treatment effect. It is also recommended that the analysis be quite conservative in recognition of the likelihood of confounding biases, eg, with regard to selection and ascertainment. Uncontrolled trials using progression-free survival or time to progression as a primary end point should be considered on a case-by-case basis, and the methodology to be applied should be thoroughly described in the protocol.

4. RESPONSE REVIEW

For trials where the response rate is the primary end point, it is strongly recommended that all responses be reviewed by an expert or experts independent of the study at the study's completion. Simultaneous review of the patients' files and radiologic images is the best approach.

(*Note:* When a review of the radiologic images is to take place, it is also recommended that images be free of marks that might obscure the lesions or bias the evaluation of the reviewer[s].)

5. REPORTING OF RESULTS

All patients included in the study must be assessed for response to treatment, even if there are major protocol treatment deviations or if they are ineligible. Each patient will be assigned one of the following categories: 1) complete response, 2) partial response, 3) stable disease, 4) progressive disease, 5) early death from malignant disease, 6) early death from toxicity, 7) early death because of other cause, or 9) unknown (not assessable, insufficient data). (*Note:* By arbitrary convention, category 9 usually designates the "unknown" status of any type of data in a clinical database.)

All of the patients who met the eligibility criteria should be included in the main analysis of the response rate. Patients in response categories 4-9 should be considered as failing to respond to treatment (disease progression). Thus, an incorrect treatment schedule or drug administration does not result in exclusion from the analysis of the response rate. Precise definitions for categories 4-9 will be protocol specific.

All conclusions should be based on all eligible patients.

Subanalyses may then be performed on the basis of a subset of patients, excluding those for whom major protocol deviations have been identified (eg, early death due to other reasons, early discontinuation of treatment, major protocol violations, etc). However, these subanalyses may not serve as the basis for drawing conclusions concerning treatment efficacy, and the reasons for excluding patients from the analysis should be clearly reported. The 95% confidence intervals should be provided.

6. RESPONSE EVALUATION IN RANDOMISED PHASE III TRIALS

Response evaluation in phase III trials may be an indicator of the relative antitumour activity of the treatments evaluated but may usually not solely predict the real therapeutic benefit for the population studied. If objective response is selected as a primary end point for a phase III study (only in circumstances where a direct relationship between objective tumour response and a real therapeutic benefit can be unambiguously demonstrated for the population studied), the same criteria as those applicable to phase II trials (RECIST guidelines) should be used.

On the other hand, some of the guidelines presented in this special article might not be required in trials, such as phase III trials, in which objective response is *not* the primary end point. For example, in such trials, it might not be necessary to measure as many as 10 target lesions or to confirm response with a follow-up assessment after 4 weeks or more. Protocols should be written clearly with respect to planned response evaluation and whether confirmation is required so as to avoid *post-hoc* decisions affecting patient evaluability
